# The anti-neuroinflammatory effects of *Clinacanthus nutans* leaf extract on metabolism elucidated through ^1^H NMR in correlation with cytokines microarray

**DOI:** 10.1371/journal.pone.0238503

**Published:** 2020-09-14

**Authors:** Amalina Ahmad Azam, Intan Safinar Ismail, Yatinesh Kumari, Mohd Farooq Shaikh, Faridah Abas, Khozirah Shaari

**Affiliations:** 1 Laboratory of Natural Products, Institute of Bioscience, Universiti Putra Malaysia, Serdang, Selangor, Malaysia; 2 Neuropharmacology Research Laboratory, Jeffrey Cheah, School of Medicine and Health Sciences, Monash University Malaysia, Subang Jaya, Selangor, Malaysia; University of Hawai'i at Manoa College of Tropical Agriculture and Human Resources, UNITED STATES

## Abstract

*Clinacanthus nutans* (CN) (Acanthaceae) is well-known for its anti-inflammatory properties among Asian communities; however, there are currently no data specifically focused on the anti-inflammatory effects of CN on the brain tissue. Neuroinflammation is a common consequence of toxin intrusion to any part of the central nervous system (CNS). As an innate immune response, the CNS may react through both protective and/or toxic actions due to the activation of neuron cells producing pro- and/or anti-inflammatory cytokines in the brain. The unresolved activation of the inflammatory cytokines’ response is associated with the pathogenesis of neurological disorders. The present study aimed to decipher the metabolic mechanism on the effects of 14 days oral treatment with CN aqueous extract in induced-lipopolysaccharides (LPS) rats through ^1^H NMR spectroscopic biomarker profiling of the brain tissue and the related cytokines. Based on the principal component analysis (PCA) of the nuclear magnetic resonance (NMR) spectral data, twenty-one metabolites in the brain tissue were profiled as biomarkers for the LPS (10 μL)-induced neuroinflammation following intracerebroventricular injection. Among the twenty-one biomarkers in the neuroinflammed rats, CN treatment of 1000 and 500 mg/kg BW successfully altered lactate, pyruvate, phosphorylcholine, glutamine, and α-ketoglutarate when compared to the negative control. Likewise, statistical isolinear multiple component analysis (SIMCA) showed that treatments by CN and the positive control drug, dextromethorphan (DXM, 5 mg/kg BW), have anti-neuroinflammatory potential. A moderate correlation, in the orthogonal partial least squares (OPLS) regression model, was found between the spectral metabolite profile and the cytokine levels. The current study revealed the existence of high levels of pro-inflammatory cytokines, namely IL-1*α*, IL-1*β*, and TNF-*α* in LPS-induced rats. Both CN dose treatments lowered IL-1*β* significantly better than DXM Interestingly, DXM and CN treatments both exhibited the upregulation of the anti-inflammatory cytokines IL-2 and 4. However, DXM has an advantage over CN in that the former also increased the expression of IL-10 of anti-inflammatory cytokines. In this study, a metabolomics approach was successfully applied to discover the mechanistic role of CN in controlling the neuroinflammatory conditions through the modulation of complex metabolite interactions in the rat brain.

## Introduction

Inflammation is a response by an immune system to either aid or remove a damaging stimulus to facilitate the healing process [1]. Inflammation signals immune cells towards the healing area, enhances blood vessel permeability, and triggers the release of inflammatory mediators [2]. Neuroinflammation coincides with peripheral inflammation in many aspects. Neuroinflammation is defined as a complex response of any aspect of brain injury which results in the activation of glial cells, and release of inflammatory mediators like cytokines and chemokines, and reactive oxygen and nitrogen species [3]. Lipopolysaccharide (LPS)-induced neurotoxicity in rats is a promising neuroinflammation study model, as LPS is a potent inflammatory agent. Through a local injection using an intracerebroventricular (ICV) technique, exogenous substances can invasively bypass the blood-brain barrier (BBB) [4] and/or increase the BBB permeability [5]. LPS induction is recognized through toll-like receptors (TLRs) in the innate immunity of its native receptors TLR-4, TLR-2, and TLR-6 [6, 7]. LPS has also been extensively used in *in vitro* experiments to induce neuroinflammation through the activation of nitrite oxidation and pro-inflammatory cytokines, such as TNF-α, IL-1*β*, and IL-6 [8, 9].

Cytokines and chemokines form a small class of signaling proteins that are crucial in coordinating the immune functions throughout the body. In the brain's immune system, this class of signaling proteins acts to maintain immune surveillance, facilitate leukocyte traffic, and recruit other inflammatory factors as they work as neuromodulators, which serve to regulate neurodevelopment and synaptic mission [10]. In a normal state, glial cells regulate innate and adaptive immune responses. However, in a disease state, activated glial cells mediate neuronal injuries through the production of pro- and anti-inflammatory cytokines, chemokines, glutamate, and reactive oxygen species (ROS) [11]. Pro- and anti-inflammatory cytokines are characterized based on their structural homology or receptors [12]. Activation of the receptor is triggered by the binding of a cytokine ligand to its cognate receptor which cascades various signalling events in cells, such as activation, adhesion, phagocytosis, cytokine secretion, proliferation, survival, death, apoptosis, and angiogenesis [13].

Extracts of the leaf material of *Clinacanthus nutans* (Burm. f.) Lindau (Acanthaceae) (CN) are a well-established therapeutic alternative for inflammation [14, 15]. Hence, the potential of CN as an anti-inflammatory agent in brain-induced inflammation was explored in this laboratory [16, 17]. A bioactivity study of CN crude aqueous extract (CNE) on nitric oxide inhibition in *in vitro* LPS-induced BV2 cells (rat microglia) revealed the extract had potential as an anti-neuroinflammatory source [16]. Nevertheless, the use of various matrices, such as cells, tissues, and biofluids offer much richer information source for metabolic profiling in direct diagnosis, therapeutic strategies, and system biology studies [18]. For the evaluating the targeted responses on pathogenesis, tissue metabolomics is deemed to be the most powerful platform as it provides direct information on metabolic modifications and upstream regulation [19].

This laboratory has previously reported on the metabolite variations in sera due to the *in vitro* perturbation following LPS and CNE treatment in a rat model [17]. A nuclear magnetic resonance (NMR)-based metabolomics approach successfully revealed the potential of CN in modulating the key differential metabolites and providing specific metabolic pathway alterations in the sera of neuroinflammed rats. Among the affected pathways were glycolysis and gluconeogenesis (lactate, glucose, and pyruvate), histidine (alanine, and histamine), lipid metabolism (acetate, ethanol, choline, and creatine), TCA cycle (citrate, and succinate), amino acid metabolism (isoleucine, leucine, and glutamate), fructose and mannose metabolism, and butanoate metabolism (3-hydroxybutyrate, and 2-hydroxybutyrate) [17]. The CNE was established to reduce acetate and choline levels significantly, while upregulating other potential key metabolites in the sera of rats in the LPS-induced neuroinflammation rat model [17]. The current research was designed with the main objective of evaluating the brain tissue derived from the same rat model to further understand the anti-inflammatory activity exerted by CNE against the LPS-induced neuroinflammation. Metabolomics was again employed in examining the chemical impact of CNE on the brain.

Based on the previous studies, including our observations [15–17, 20], the use of a robust analytical technique, such as NMR spectroscopy in a metabolomics approach, provides an information-rich environment for fingerprinting the potential bioactive metabolites. The pairing of NMR analysis with multivariate statistical methods is useful in the identification of biomarker(s) in a certain metabolic status [14]. Thus, the metabolomic analysis of the ^1^H NMR brain tissue data has provided insights into the CN therapeutic response and its possible mechanistic pathways. Notably, the analysis revealed the close relationship between neuroinflammation and cytokines activation, as described herein.

## Materials and methods

### Chemicals and reagents

The NMR reagents used for measurements, namely deuterium oxide (D_2_O, 99.9%), deuterated methanol (CD_3_OD, 99.9%), deuterated sodium hydroxide (NaOD), trimethylsilylpropionic acid-*d*_*4*_ (TSP), dextromethorphan hydrobromide (C_18_H_25_NO.HBr, 99%), and potassium dihydrogen phosphate (KH_2_PO_4_), were purchased from Merck (Darmstadt, Germany). Lipopolysaccharide (LPS) injection derived from *Escherichia coli* 026: B6, sterile-filtered phosphate buffer saline (PBS), dextromethorphan hydrobromide (DXM), and sterile purified water were obtained from Sigma Aldrich (St. Louis, MO, USA). Absolute methanol (MeOH) and chloroform (CHCl_3_) were procured from Fisher Scientific UK (Leicester, UK). Ultrapure distilled water was prepared using a Milli-Q purification system. The normal rat chow feed was purchased from Specialty Feeds (Glen Forrest, Australia). The Pierce^®^ 660 nm Protein Assay reagents were supplied by Thermo Scientific (Waltham, MA, USA), and bovine serum albumin (BSA, 99% pure grade) was purchased from Sigma Aldrich (St. Louis, MO, USA). The complete kit for cytokines quantification; G-series Rat Inflammation Array 1; catalog no: GSR-INF-1 was purchased from RayBiotech (Norcross, GA, USA).

### Plant collection and extraction

*Clinacanthus nutans* (CN) plants, grown under the same environmental and growth conditions, were collected in December 2015 at Sendayan, Negeri Sembilan (GPS coordinates: 4.52° N, 100.48° E), Malaysia. Plant authentication was carried out by a botanist, Dr. Shamsul Khamis, and a voucher specimen (SK 2883/15) was deposited at the Unit Herbarium of Biodiversity, Institute of Bioscience, Universiti Putra Malaysia. The leaves were collected by separation from the stems, then cleaned and dried under shade for nine days at room temperature (27 to 30°C). The dried leaves were ground to a powder in a blender, and uniformity in size was confirmed by sieving through a stainless-steel mesh of 200 mm diameter with a nominal aperture size of 500 micrometers. The CN leaf powder was stored in airtight containers at 3 ± 2°C prior to extraction. The leaf material was extracted for three days at the room temperature in the dark by immersion in a ratio of 1 g plant material: 50 mL of ultrapure distilled water. The resulting extract was filtered before repeating the extraction twice with fresh ultrapure distilled water. The pooled CN extracts were concentrated by removal of the solvent using a rotary evaporator at 40°C. The extraction method is the same as the published report on CNE by this laboratory [16, 17], which was slightly modified from the procedure of Khoo and colleagues [15, 21]. The lyophilized CN crude extract (CNE) (extraction yield; water: 30% w/w of the actual percentage of dry leaf weight) was kept frozen until further use.

### Animal husbandry and sample collection

All of the animal experiments with thirty-five, thirteen-week old male Sprague Dawley (SD) rats (300 ± 50 g) were carried out at the housing complex of the Laboratory of Animal Resources, Universiti Kebangsaan Malaysia (UKM) (Bangi, Malaysia) which complies with the Malaysian regulations on animal welfare guidelines and were approved by the Universiti Putra Malaysia Animal Ethics Committee (Approval number: UPM/IACUC/AUP189 R070/2015). The rats were purchased from the in-house breeding program of the same Laboratory of Animal Resources, UKM. Animals were housed in polycarbonate cages and kept under controlled conditions (light/dark cycle, 12/12 h; temperature, 24±2°C) and acclimated for a week prior to the experiments. The rats had unrestricted access to water and a standard rodent diet. LPS or phosphate buffer saline (PBS) was injected through ICV, whereas water, CN extracts, and DXM were administered by oral gavage. The rats were then randomly divided into the following seven groups as shown in Table 1 below.

**Table 1:** Grouping of rats according to treatment.

Group	ICV Induction	Treatment	Definition
**N+water (n = 5)**	PBS, 10 μL	Water	Normal control
**N+500CN (n = 5)**		CNE at 500 mg/kg BW	Normal treated control with CNE, 500 mg/kg of BW
**LPS+water (n = 5)**	LPS, 1 μg/1μL	Water	Neuroinflammed control rats
**LPS+250CN (n = 5)**		CNE at 250 mg/kg BW	Neuroinflammed rats treated with CNE, 250 mg/kg BW
**LPS+500CN (n = 5)**		CNE at 250 mg/kg BW	Neuroinflammed rats treated with CNE, 500 mg/kg of BW
**LPS+250CN (n = 5)**		CNE at 1000 mg/kg BW	Neuroinflammed rats treated with CNE, 1000 mg/kg of BW
**LPS+DXM (n = 5)**		DXM at 5 mg/kg BW	Neuroinflammed rats treated with dextromethorphan, 5 mg/kg of BW as the positive control

PBS = phosphate buffer saline; LPS = lipopolysaccharides; CNE = *Clinacanthus nutans* aqueous extract; BW = body weight of rat; and n = total number of rats per group.

The doses for administration of the CN extract were selected according to Lau and colleagues with slight modifications [22]. The induction of either 10 μL phosphate buffer saline (PBS) to the normal rat groups or lipopolysaccharides (LPS, 1 μg/1μL) to the neuroinflammed groups have been described elsewhere [17]. In brief, the rats were anesthetized with ketamine-xylazine (K-X); K: 80 mg/kg BW; X: 10 mg/kg of BW through the intraperitoneal (i.p.) route, and underwent stereotaxic surgery after positioning on a stereotaxic frame. A midline incision of the scalp was made, and the vertex area was exposed. A single injection of a solution of either LPS (10 μL, 1 μg/1 μL) freshly dissolved in PBS, or PBS alone filtered through a 0.22 μm membrane filter was injected through ICV into the location of the substantia nigra on the right side of the small drilled hole according to coordinate relative to bregma: anterior-posterior (AP) = -5.5mm, lateral-medial (LM) = +1.8 mm; dorsal-ventral (DV) = -8.3 mm (location of substantia nigra at right side of the brain) with a consistent rate of 3 μL per minute using a Harvard Apparatus Pump 11 elite infusion syringe through a Hamilton syringe (Holliston, MA, USA).

One week after the injection, the rats were administered, once daily for two weeks by oral gavage, with either the stock solution of CNE (250 mg, 500 mg, or 1000 mg/kg BW), normal water (1 mL), or a 5 mg/kg bw dose of DXM for fourteen consecutive days. CNE stock extracts were preserved at 4°C and used within three days, while DXM was freshly prepared prior to use. Each of the rats was euthanized under anesthetization of K-X with the terminal process through exsanguination by cardiac puncture. The serum was analyzed according to an earlier published report from this laboratory [17].

The whole-brain tissue was harvested and cleaned with cold PBS. The brains were kept in dry ice for 1 minute before further excisions. From the dorsal view, the brain was horizontally cut as thick as 5 mm at the coordinate 5–10 mm above the area of cerebral transverse tissue from the substantia nigra area. Part of the substantia nigra around 4 mm^2^ from both sides of the brain was collected and pooled into a microcentrifuge tube to be used for the cytokine analysis. The remainder of the brain tissue was collected into separate vials and used for the ^1^H NMR metabolomic profiling studies. All of the excised animal parts were kept under -80°C prior to use.

### ^1^H NMR spectroscopy of brain tissue

The low molecular weight, water-soluble components were extracted from the homogenized brain samples using CHCl_3_/MeOH/H_2_O (2/1/1 v/v/v) according to the Folch method [23], with modifications. Briefly, CHCl_3_/MeOH (2:1, 12 mL) was added to a brain homogenate of 500 mg wet brain/mL of saline solution and vortexed for 15 minutes. After 1 h standing at room temperature, distilled H_2_O (3 mL) was added to solubilize the hydrophilic components. The mixture was centrifuged at 112 x g for 1 h before the water-soluble phase was separated from the CHCl_3_ phase. The water-soluble phase (4 mL) of each sample was freeze-dried for 8 h and stored at -80°C until analysis.

Each of the thirty-five dried samples was dissolved in a phosphate buffer solution containing 0.2% trimethylsilylpropionic acid sodium salt (TSP) in D_2_O (pH, 7.4) (600 μL) and transferred into a 5 mm standard NMR tube (Norell, Sigma-Aldrich, Canada). The NMR spectra were analyzed according to a published protocol [24] using a standard one dimensional (1D) ^1^H NMR spectroscopic technique at 500 MHz (Varian Inova 500, IL, USA). In brief, the experiment was performed at 25˚C with the parameters of pulse width (PW) 21.0 μs (90°) and a relaxation delay (RD) of 2.0 s. Suppression of the water signal in the pre-saturation sequence was used first to suppress the residual water with low power selective irradiation, then a transverse relaxation time of T2 measurement Carr-Purcell-Meiboom-Gill (CPMG) was performed using the following parameters: inter-pulse delay (τ) of 0.0004 s and big τ (eighty 180-degree refocusing pulses) of 0.8 s; relaxation delay (RD) 0.5 s with a transient of 128 scans.

#### Quantitative cytokines measurement in brain tissue

Cytokine expression levels of the brain protein lysate samples were measured using the G-series rat inflammation array (GSR-INF-1, RayBiotech, Inc., Norcross, GA, USA) [25]. The microarray is a commercialized rat-specific, a multi-spot plate of various Th1/Th2 cytokines: interferon (IFN)-*γ*, interleukin (IL)-1*α*, IL-1*β*, IL-2, IL-4, IL-6, IL-10, IL-13, monocyte chemoattractant protein 1 (MCP-1), and tumor necrosis factor (TNF)-*α*. The concentrations of each cytokine were arrayed in quadruplicate, together with positive and negative controls. The sample proteins were quantified and standardized using the Pierce® 660 nm Protein Assay at a predetermined concentration of 500 μg/mL protein lysate in the supplied RIPA buffer obtained from the complete kit of G-series Rat Inflammation Array 1; catalog no: GSR-INF-1 (Ray Bio^®,^ Norcross, GA, USA). This was to generate a 6-point series BSA standard curve as conducted previously [26, 27]. The procedure was performed according to the manufacturer’s protocol with blocking and incubation of sample diluent in 30 min at room temperature, overnight sample incubation at 4°C, treatment with 80 μL biotin-conjugated antibodies for 2 hours, and washing with the kit-supplied buffers. The array was also dyed with cy3-streptavidin and let stand for 1 h before the fluorescent signals were visualized using an Axon GenePix 4300A laser scanner (Molecular Devices, Sunnyvale, CA, USA) at 532-nm excitation. Data were extracted with RayBio Q analyzer software (RayBiotech, Inc., Singapore) and the spot signal intensities for antigen-specific antibody between groups were utilized to determine the relative differences in expression levels of each sample after subtraction of background and normalization to positive controls [28].

### Statistical analysis of quantitative cytokine levels and univariate statistical analysis of NMR spectral data

The quantitative data of cytokines were obtained from the microarray results, and the quantitative data of the metabolites were derived from the NMR spectral bins. One-way analysis of variance (ANOVA) with GraphPad Prism V 7.0 (GraphPad Software Inc, San Diego, CA, USA) was used to interpret the data. Tukey’s test was applied to determine the difference within the groups. The results were displayed as mean ± standard error of means (SEM) in which a p-value < 0.05 was considered significant.

### NMR spectral data processing and multivariate data analysis

The raw ^1^H NMR spectral data were manually phased, baseline corrected, and referred to the internal standard (TSP) at 0.00 ppm. The integrated bins of 0.04 ppm width for the chemical shift (δ) region from 0 to 10 ppm were reduced using the Chenomx NMR software package (Chenomx NMR Suite 5.1 Professional, Edmonton, Alberta, Canada) before further analysis. The region associated with residual water (4.66–5.05 ppm) was removed and the resulting spectral segments for each NMR spectrum were normalized to the total sum of the spectral intensity, which partially compensated for the difference in metabolite concentration between each sample. NMR data were then subjected to multivariate statistical methods with statistical isolinear multiple component analysis (SIMCA)-P 13.0 software package (Umetrics, Umeå, Sweden) for pattern recognition. The binned data were mean-centered, and Pareto scaled before performing Principal Component Analysis (PCA) and Orthogonal Partial Least Squares-Discriminant Analysis (OPLS-DA). The scores plot consisted of two principal components (PC1 and PC2) in which each point on the plot represented an individual spectrum of a sample. The score plot visualizes the observation of the groups’ cluster patterns. The group separation associated with the metabolites was indicated by the corresponding loading plots, where each point represented a single NMR spectral bin. The Hotelling’s T^2^ has a cumulative score of each component in the PC or PLS model, whereby T2 measures how far an observation is from the center of the model. This is a multivariate generalization of Student’s T-test which provides a check for multivariate normality among the observations [29]. In conjunction with a two-dimensional score plot, a confidence ellipse is represented based on the Hotelling's T^2^, as it defines the normal area corresponding to the confidence interval. In the present study, a significance level 0.05 or at 95% confidence was used. Thus, observations situated outside the ellipse could be considered as outliers [29]. The validation and significance of the model were performed using a three-fold method of 100 permutation tests, and the calculation of R2Y and Q2Y values.

### Integrative data matrices and pathway analysis

The integrative work set of data matrices between metabolites and cytokine quantification analysis was normalized using transformed page criteria in SIMCA ver.13 software. The pathway was generated using a web tool of Integrated Molecular Pathway Level Analysis, IMPaLA (http://impala.molgen.mpg.de/), which is a freely available, web-based platform able to perform the integrative, over-representation analysis.

## Results and discussion

**Fig 1:**
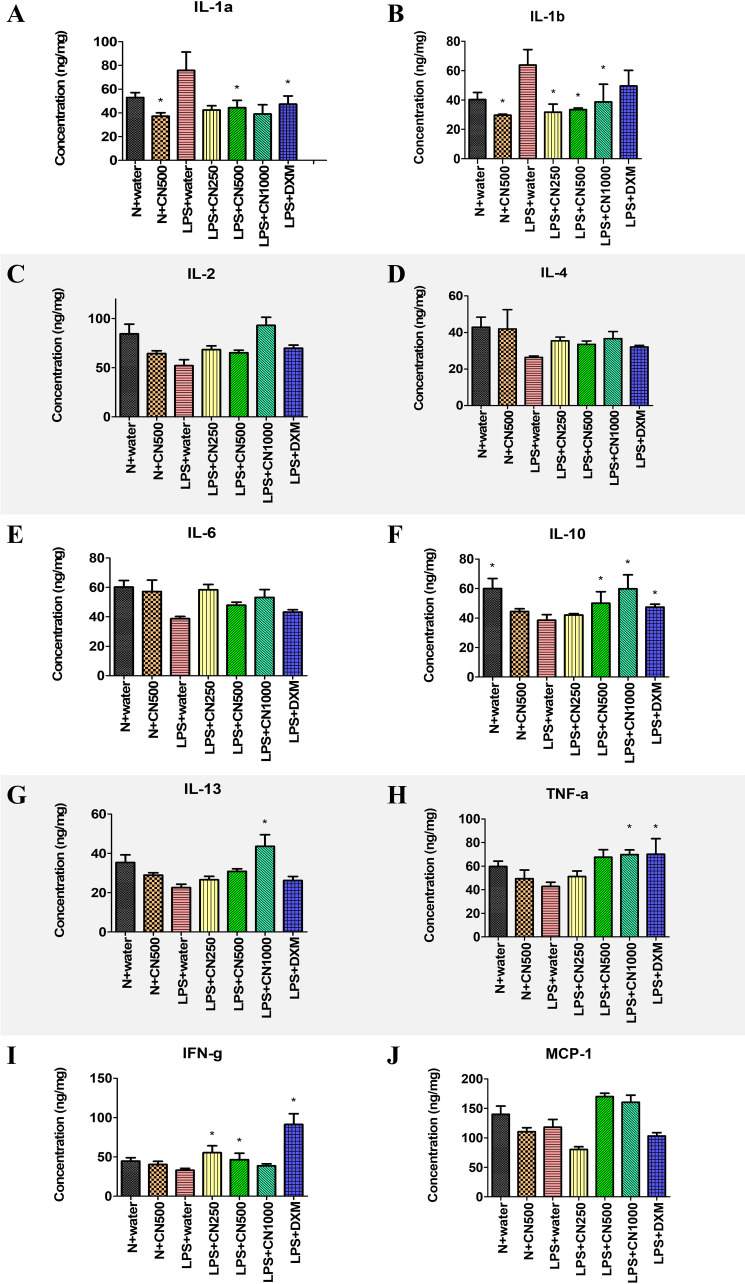
The cytokines (A-I) and chemokine (J) concentrations (expressed as mean±SEM) in brain tissue inflammation after 14 days of treatment observed through microarray. Data are expressed as mean ± standard error of mean (SEM) and analyzed by one-way ANOVA, followed by Tukey’s test. Whereby *p<0.05 shows a significant difference as compared to LPS+water.

**Fig 2:**
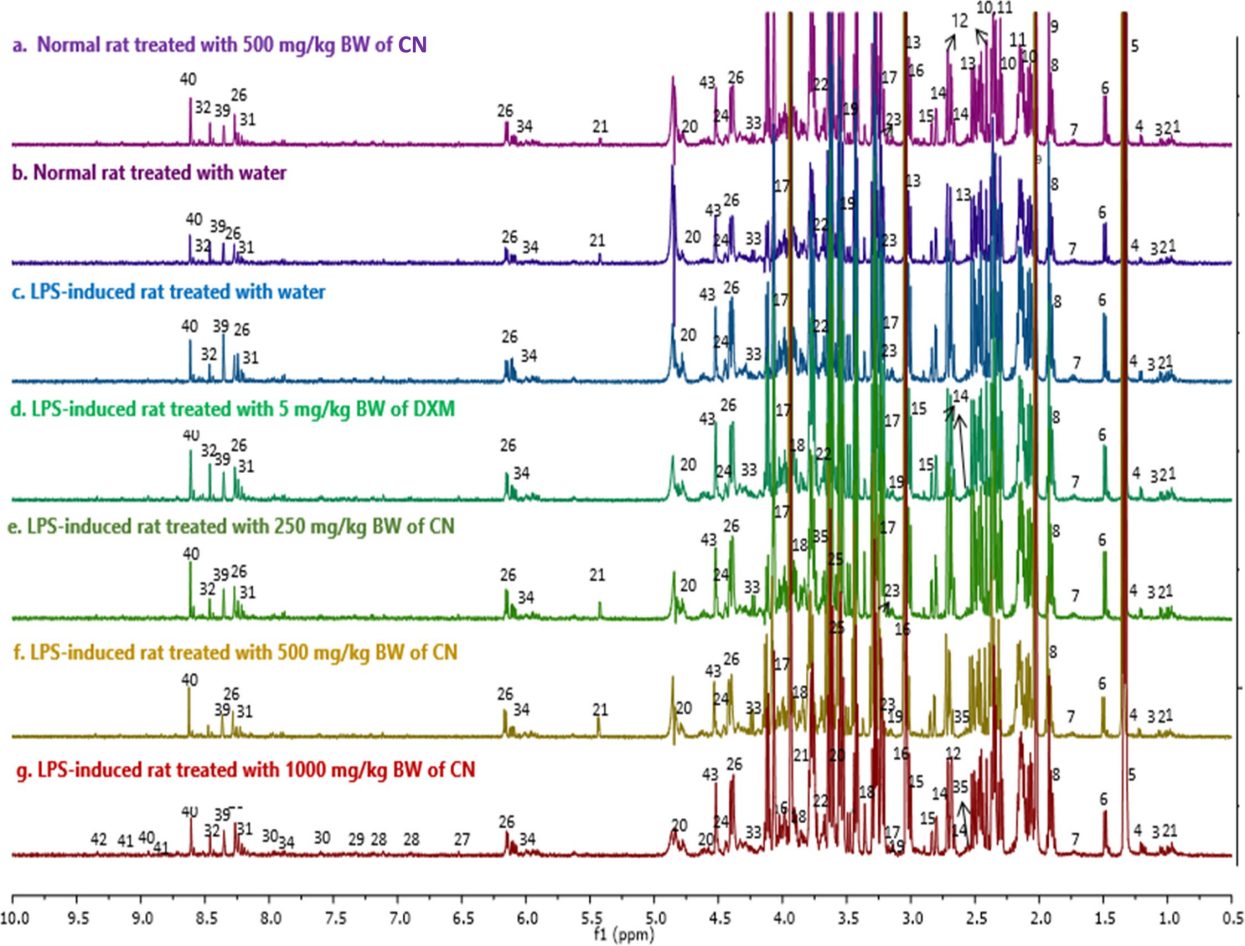
^1^H NMR representative spectra of the brain tissue with putative and tentative metabolites. (a) normal rats treated with water, (b) normal rats treated with 500 mg/kg BW of CN, (c) LPS-induced rats, (d) 5 mg/kg BW of DXM, (e) 250 mg/kg BW of CN, (f) 500 mg/kg BW of CN, and (g) 1000 mg/kg BW of CN after 14 days treatment. Identified metabolites: (1) isoleucine, (2) leucine, (3) valine, (4) 3-hydroxybutyrate, (5) lactate, (6) alanine, (7) lysine, (8) GABA, (9) acetate, (10) glutamate, (11) glutamine, (12) malate, (13) *α*-ketoglutarate, (14) citrate, (15) aspartate, (16) creatine/phosphocreatine (17) choline, (18) myo-inositol, (19) taurine, (20) *β*-glucose, (21) *α*-glucose, (22) glycerol, (23) phosphorylcholine, (24) dihydroxyacetone, (25) glycine, (26) inosine, (27) fumarate, (28) tyrosine, (29) phenylalanine, (30) nicotinurate, (31) hypoxanthine, (32) formate, (33) threonine, (34) UDP/UTP, (35) pyruvate, (36) histidine, (37) succinate, (38) serine, (39) 2-deoxyadenosine, (40) nicotinate, (41) trigonelline, (42) NADP+, and (43) anserine.

**Fig 3:**
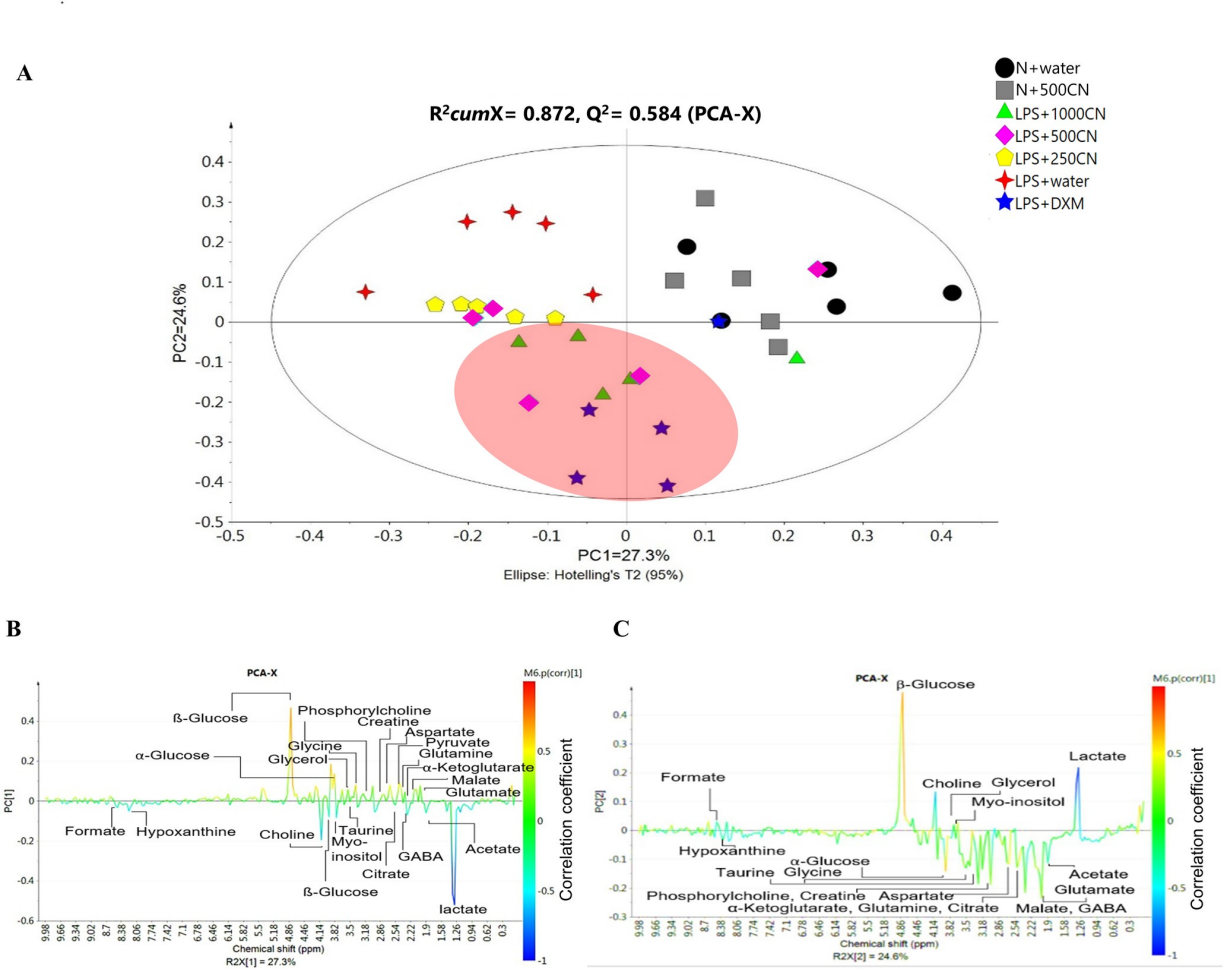
Differentiation of a pairwise comparison in the ^1^H NMR spectra of the rat brain tissue samples after 14 days of CNE treatment. (A) PCA score plot, (B) the loading line plot derived from PC1, and (C) PC2. (A) represents the score plot of the PCA model, with a validated metabolomics model, R^2^*cum*X = 0.872, Q^2^ = 0.584, with Ellipse Hotelling’s T^2^ at 95%. All the points are inside the elliptical region, meaning that all of the observations have fulfilled at least 95% adherence to the statistical multivariate normality. Generally, Hotelling T^2^ is a diagnostic tool to show outliers. Thus, no outlier is detected in this model. Figures (B) and (C) represent colour-coded coefficient loading line plots for the PCA model of ^1^H NMR brain tissue metabolic profile for normal vs neuroinflammed rats by PC1, and between treated rat with CNE/DXM vs control group by PC2. Symbols of the black circle, grey square, green triangle, pink diamond, yellow pentagon, four-point star in red and five-point star in blue represent the N+water, N+500CN, LPS+1000CN, LPS+500CN, LPS+250CN, LPS+water, and LPS+DXM treatment groups, respectively. Twenty-one potential key metabolites for both class separations were labeled accordingly to their resonances in the NMR spectrum (ppm).

**Fig 4:**
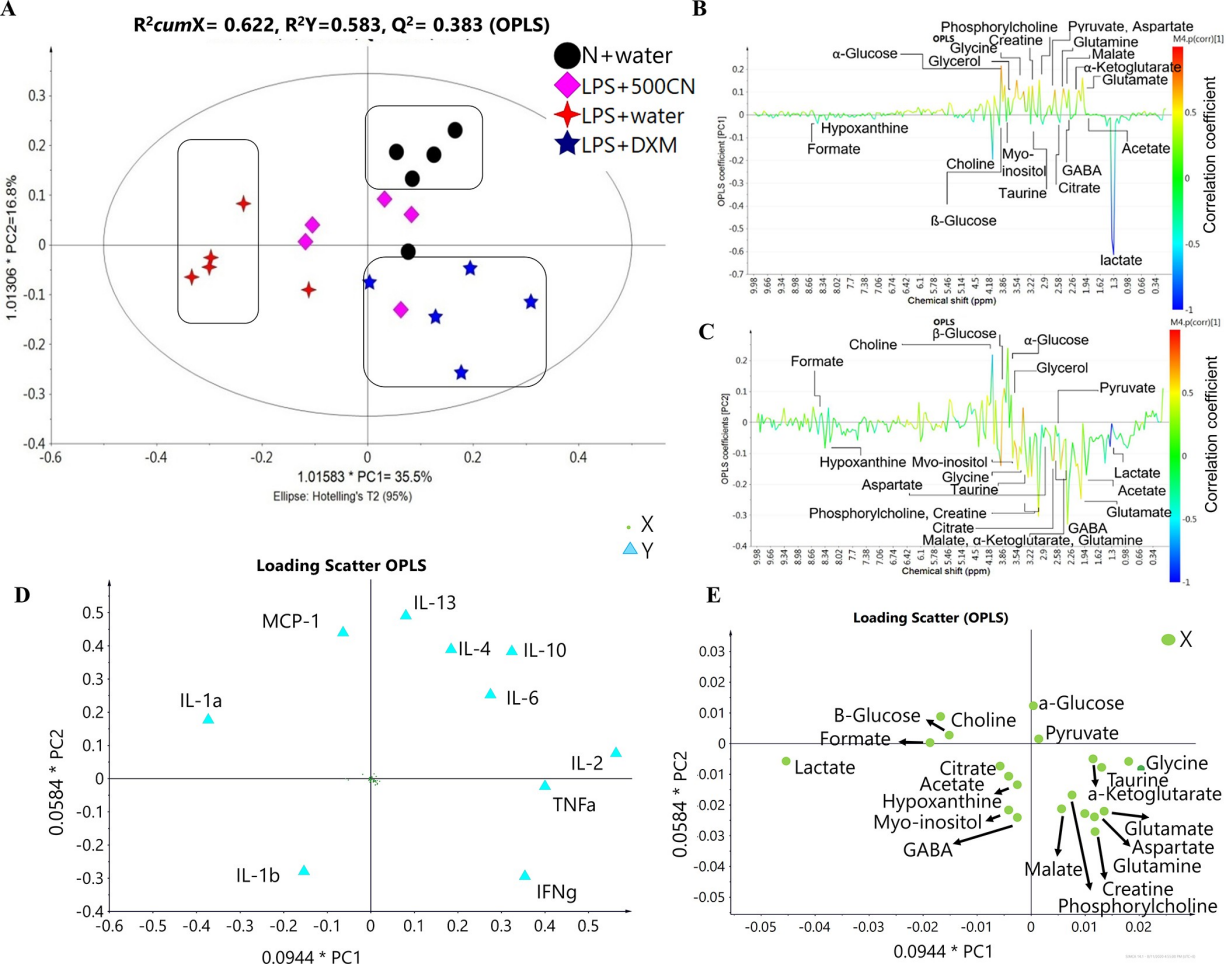
Differentiation of a pairwise comparison on the ^1^H NMR spectra of the rat brain tissue samples after 14 days of CNE treatment. (A) OPLS score plot, (B) the loading line plot derived from PC1, and (C) PC2, (D) scatter plot based on cytokines expression, and (E) metabolites. (A) represents the score plot for the OPLS model, with validation of R^2^*cum*X = 0.622, R^2^Y = 0.583, Q^2^ = 0.383, which variable to be explained at 35.5% (PC1) and 16.8% (PC2). The Ellipse Hotelling’s T^2^ is limited at 95% confidence, which is the ellipse represented in the plot. All the points are inside the elliptical region. Thus, no outlier is detected in this model. (B) and (C) represent colour-coded coefficient loading line plots for the OPLS model of ^1^H NMR brain tissue metabolic profiles of normal rats, LPS treated with CN and DXM vs neuroinflammed rats for principal component 1 (PC1), and between LPS treated rat with CNE vs DXM for principal component 2 (PC2). Twenty-one potential key metabolites for both class separations were labeled according to their resonances (ppm) in the NMR spectrum. (D) and (E) are loading scatter plots of the same model visualized pattern distribution for the *X* and *Y* variables with 0.0944*PC1 and 0.0584*PC2 coefficient correlation. (D) shows the *Y*-variables distribution, however, the *X*-variables are too small to be seen. Thus, the scale for the metabolite (*X* variables) distribution was increased in (E) for better visualisation. Symbols of the black circle, pink diamond, four-point star in red, and five-point star in dark blue represent the N+water, LPS+500CN, LPS+water, and LPS+DXM treatment groups, respectively, whereas the green circle is for the *X* variables/^1^H NMR metabolites and light blue triangle is for the *Y* variables/cytokines and chemokines expression.

**Fig 5:**
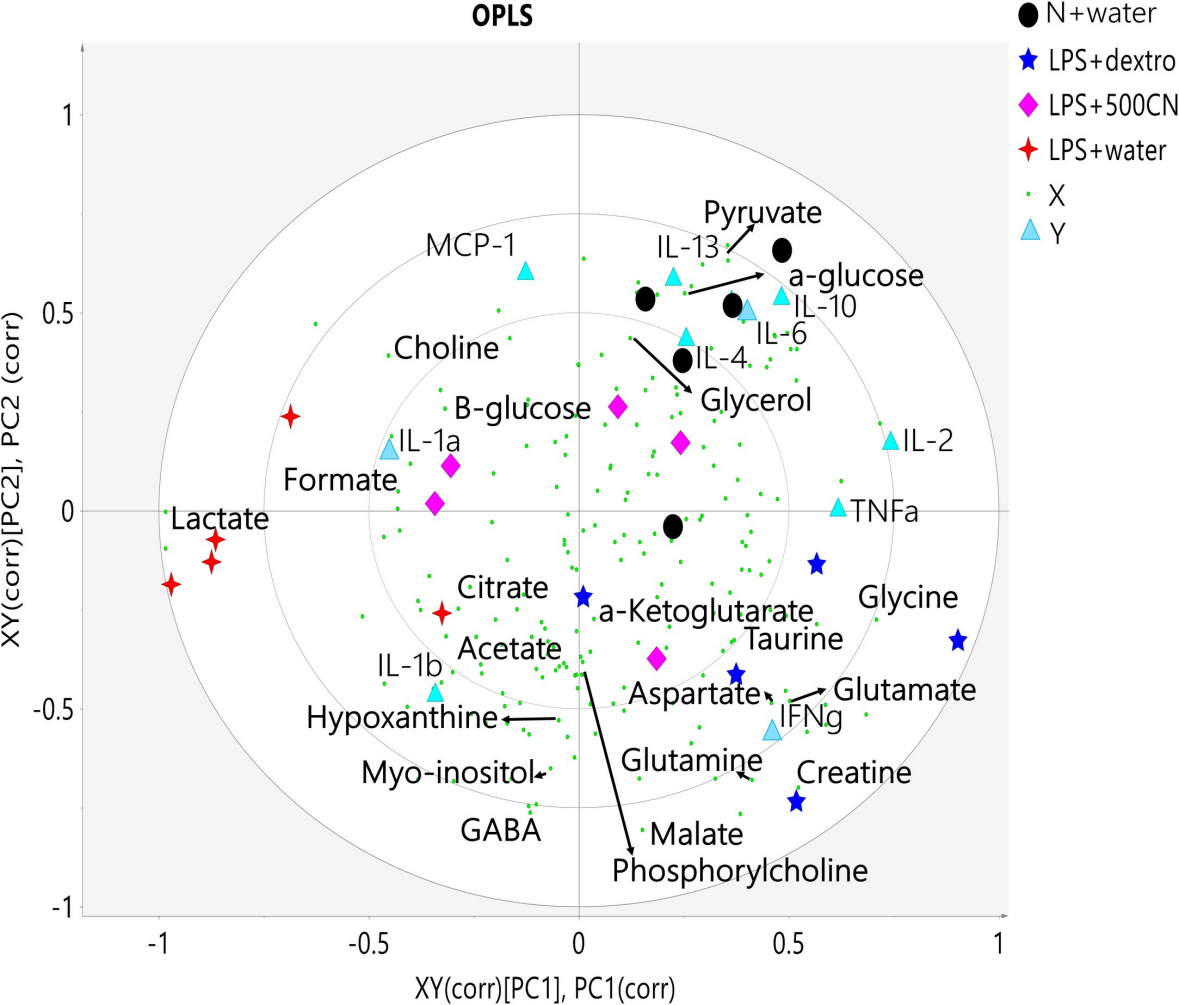
Biplot of the OPLS model. Fig 5 captures a combination plot between the score plot and the loading scatter plot (Fig 4) with each principal component (PC) correlated to the *X* and *Y* variables, XY(corr)[PC1], PC1(corr) at PC1, and XY(corr)[PC2], PC2(corr) at PC2. Symbols of the black circle, pink diamond, four-point star in red, and the five-point star in dark blue represent N+water, LPS+500CN, LPS+water, and LPS+DXM treatment groups, respectively, whereas the small green circle represents *X* variables/^1^H NMR metabolites and the light blue triangle indicates the Y variables/cytokines and chemokine levels.

The quantitative measurement of cytokines and chemokines in the brain tissue of rats, as shown in Fig 1, revealed complications in understanding and interpreting the regulatory pattern of pro- and anti-neuroinflammatory activity of CN treatment. A ^1^H NMR-based metabolomics approach was applied to assist in the assessment of the anti-inflammatory potential of CN (Fig 2). PCA analysis of the ^1^H NMR metabolite profile of the brain tissue (Fig 3) revealed the affected metabolic pathways based on both the potential biomarkers of LPS-induced, neuroinflammed condition, and the metabolite alterations caused by CN intervention. The ameliorative activity of CN was successfully exhibited through integration of the data of the cytokine microarray in the OPLS model, providing a better explanation regarding the variation in the cytokines (Figs 4 and 5). Comprehensive analysis of the CN extracts provided insights into the association between the metabolites and the cytokine expression at the network level.

### Phytochemical analysis of the CN aqueous extract

Identification of the phytochemical constituents in the CN aqueous extract (CNE) through ^1^H NMR spectral analysis was previously reported by this laboratory [15–17, 21]. The qualitative and quantitative identification of most of the metabolites present in CNE was comprehensively accomplished by ^1^H NMR, and the presence of some selected metabolites was established by HPLC through comparison with standards [15]. Putative identification revealed forty-four metabolites including triterpenes, sulfur-containing glycosides, *C*-glycosyl flavones, and a variety of other compounds including sugars, amino acids, and phenolics [15–17, 21]. The present study of CNE showed a similar ^1^H NMR spectroscopic pattern [15], with a high intensity of the shaftoside resonance [16, 17]. In our recent report on CNE phytoconstituents with NO inhibitory activities in the BV2 cell assay revealed 30 key chemical markers, namely schaftoside, acetate, propionate, alanine, clinacoside C, monoacyl-monogalactosylglycerol, fructose, clinacoslides A and B, ascorbic acid, a mixture of cerebrosides, choline, stigmasterol-*β*-glucoside, citric acid, valine, catechin, orientin, chlorogenic acid, leucine, butyrate, cycloclinacosides A1 and A2, sucrose, vitexin, *β*-sitosterol, *β*-glucose, vanillic acid, gendarucin A, betulin, and isoleucine [16]. Khoo *et al*. also successfully demonstrated through HPLC-DAD-ESI-MS/MS quantification that four metabolites, namely shaftoside (0.65±0.03 mg/g), isovitexin (0.128±0.007 mg/g), orientin (0.005±0.00 mg/g), and isoorientin (0.004±0.000 mg/g), were present in the CNE, wherein all these four constituents were also identified in the current study [15, 16].

### Immune response to LPS-induced neuroinflammation and CN intervention observed through application of the cytokine microarray

Cytokines are signaling protein molecules, commonly less than 80 kDa in size, which function biologically at picomolar or nanomolar concentrations. In addition to their integral roles in inflammation regulation and the modulation of cellular activities [30], they participate in sleep regulation, neuroendocrine functions, neuronal development, and normal aging [31]. This diverse group of cytokines includes numerous interleukins (ILs), lymphokines, chemokines, hematopoietins, interferons (IFN), platelet-derived growth factors (PDGF), transforming growth factors (TGF), and the tumor necrosis factor (TNF) families.

Cytokine levels are normally low, but they can markedly increase in response to an endogenous pathogen, inflammation, or through tissue injury. As the principal aim of this study was concerned with understanding cytokines as mediators of altered function of the central nervous system (CNS) during the intervention of a pyrogenic component of Gram-negative bacteria called LPS, the focus was on the ILs (IL-1*α*, IL-*β*, IL-2, IL-4, IL-6, IL-10, IL-13), TNF-*α*, IFN-γ, and chemokine, namely MCP1, which is also known as chemokine ligand 2 (CCL-2). The photographs of the scanned microarray are presented in S1 Fig A in S1 File. Fig 1 shows the signal quantification data of protein expression, after 14 days of treatment, for the concentration of the ten selected cytokines and a chemokine in the substantia nigra brain tissue.

The cytokines were divided into pro- and anti-inflammatory factors, which are commonly characterized based on their structural homology or their target receptors [12]. In the current research, cytokines were categorized into pro- and anti-, and the synergistic functions of the pro- and anti-actions were considered [32]. However, it is recognized that understanding the exact properties of any given cytokine and the determination of its grouping is a challenge. There is unequivocal evidence that cytokines are often pleiotropic in their effects. Cavaillon [33] pointed out that the classification of cytokines could change in response to the nature of the target cell, the activating signal, the timing of the sequence of cytokine action, and the experimental model.

Interleukins IL-1*α* and IL-1*β* are the key regulators of LPS inflammation [34]. In Fig 1A, there are significant levels of down-regulated IL-1*α* expression between the LPS-induced neuroinflammed and the groups treated with CN and DXM. This suggests that CN was able to reduce the pro-inflammatory cytokine IL-1*α*. Yet, the level of expression between the normal control group (N+water) failed to show a significant difference when compared to the LPS+water only. Hence, the utility of this model is debatable. In Fig 1B, all of the LPS-induced groups expressed more of IL-1*β* than the normal (N) groups. A significant reduction could be seen in all of the CN treatments regardless of the dose. However, the LPS+DXM (positive control drug) also showed an insignificant reduction in the cytokines’ expressions. Although both IL-1s have shown significant reduction in their expressions after CN treatment, the overall pattern was not clear. The other pro-inflammatory mediators, such as IL-6 (Fig 1E), have increased in the normal rats, as well as in all of the LPS-induced rats, regardless of any treatment when compared with LPS+water. However, these changes were without any significant differences.

The other pro-inflammatory cytokines of TNF-*α* and IFN-*ϓ* [9], in (Fig 1H and 1I, respectively), showed up-regulation in both expressions for all LPS-induced rats treated with CN and DXM when compared with the LPS control. These observations established that the treatments were not effective in inhibiting the pro-inflammatory cytokines. However, the expression of IL-10, an anti-inflammatory cytokine in the LPS mechanism [9], was significant for the LPS+CN 500 and 1000 mg/kg of BW groups, and the positive control group (LPS+DXM). This proved that there was a positive impact by both treatments of CN and DXM. Nevertheless, other cytokines, such as IL-2, IL-4, and MCP (Fig 1C, 1D and 1J, respectively), were insignificantly up- regulated when compared with the LPS control group. MCP-1, as in Fig 1J, is one of the key chemokines that regulates the migration and infiltration of monocytes/macrophages in response to inflammation [35]. However, in this study, MCP-1 was lower in the LPS+water group compared to the normal control. This might be due to the hypoxia condition induced by the accumulation of lactate, choline, and acetate (indicated by red arrow) in the PCA loading scatter plot of the ^1^H NMR brain tissue (S2 Fig B in S1 File). Hypoxia has been proven to reduce the constitutive MCP-1 expression at the mRNA and protein levels in human proximal renal tubular cells [36]. This represents unequivocal evidence that cytokines are often pleiotropic in their effects [33].

For a holistic view, using the microarray results alone it was difficult to interpret the regulatory pattern of the cytokines and chemokine. Simplification of the cytokine action studies was also proposed by O’Shea *et al*. [37] using the generation of gene-targeted mouse model. As an alternative to the gene model, a validated animal model using a metabolomics approach is one of the best choices [38]. The profiling of diseases in an animal model through the integration of cytokine data with spectroscopic data using a metabolomics approach has been established [39–41]. Hence, the accumulated ^1^H NMR brain tissue data were correlated with the response of ten specific cytokines and a chemokine for a better interpretation of the CNE intervention.

### Metabolic characterization of LPS-induced neuroinflammation rats

The representative ^1^H NMR spectra of the rat brain tissue obtained from the seven study groups: the LPS-induced rats treated for 14 days with 1000, 500, and 250 mg/kg BW of CN, the LPS-induced rats treated with 5 mg/kg BW of DXM, the LPS-induced rats, the normal rats treated with 500 mg/kg BW of CN, and the normal rats, are shown in Fig 2. Forty-three metabolites which characterized the various groups were identified based on the compound library of Chenomx NMR suite 5.1 professional (Chenomx Inc., Edmonton, Canada) using the peak fitting method, and through comparison with the reported 1D ^1^H NMR chemical shifts in the literature and in accessible metabolomic databases, such as HMDB (http://www.hmdb.ca), METLIN (http://metlin.scripps.edu), and KEGG (http://www.kegg.jp). The metabolite characterizations were then confirmed by *J*-resolved NMR and two-dimensional HMBC (^1^H-^13^C Heteronuclear Multiple Bond Correlations) NMR. S3 Table C in S1 File summarizes the assigned and identified putative markers in the NMR spectral brain tissue of rats with a tolerance of ±0.02 ppm for ^1^H NMR, ±10 Hz for *J*-resolved and ±0.5 ppm for ^13^C NMR.

Visual inspection of the spectra (Fig 2) could not indicate clear metabolite changes. Thus, multivariate data analysis was adopted to clarify and detect the metabolite changes in the brain tissues that occurred after the chemical intervention of LPS and 14 days of CN and DXM treatments. A supplementary figure of four selected spectra of normal rats injected with PBS + water as control (N+water), LPS-neuroinflammed rats + water as control (LPS+water), LPS-neuroinflammed rats treated with aqueous CN at 500 mg/kg of BW (LPS+500CN), and neuroinflammed rats + dextromethorphan (LPS+DXM) at different intensity ranges from Fig 2 was also extracted for enhanced visualization view. In S4 Fig D in S1 File, the twenty-one, selected comparable metabolites peak intensities of either the normal or the LPS-induced control rats were labeled.

In the PCA score plots (Fig 3), the X- and Y-axis represent PC1 and PC2, respectively, and each point represents a sample from an individual rat. The spectral data of the brain tissue from the rats of the LPS-induced groups 3, 4, 5, 6, and 7 derived after 14 days of treatment with either CN, DXM, or water were biochemically distinct from those of the normal rats injected with PBS (groups 1 and 2). The criteria of a good metabolomics model are based on the internal cumulative cross-validation of the goodness of fit (R^2^*cum*) and good predictive ability (Q^2^), wherein R^2^ must be larger than Q^2^, while the Q^2^ value is higher than 0.5 [29]. The discrete clusters were observed along PC1 (Fig 3A) of a validated metabolomics model with R^2^*cum*X = 0.872, Q^2^ = 0.584. This score plot shows that the treatment with CN of 500 and 1000 mg/kg BW provided a positive effect which was possibly an amelioration similar to that provided by the DXM (5 mg/kg BW) treated group, albeit at a very significantly higher dose.

The LPS+500 and +1000 mg/kg BW of CN groups and the DXM group were separated by PC2 from the LPS+water and the lower dose of 250 mg/kg BW of CN groups. The loading line plots based on PC1 and PC2 of this PCA model, as shown in Fig 3B and 3C, respectively, provide the metabolite variations color-coded according to the value of the correlation coefficients. The correlation coefficient determines the degree to which two variables are associated. The range of value for the correlation coefficient is -1.0 (blue) to 1.0 (red). When the correlation coefficient is greater than zero, the variables have a positive relationship, and vice versa when the value is less than zero. A value of zero indicates there is no relationship between the two variables [29]. In PCA, the variables are correlated between the N rows of observation (sample treatment) and the K column of variables (metabolites) [42]. In the loading line plots, the upward hot-coloured red signal indicates the higher concentration density corresponding to the class separation than a cold-coloured blue. The hot colour signal (red) indicates a more significant contribution than the cold colour signal (blue). The upward-oriented peaks represent the positive quadrants of PC1, which correlate to the normal rat groups, and the opposite quadrants denote the LPS neuroinflammed groups (Fig 3A). In Fig 3C, the separation by PC2 exhibits the upward peaks for control of the normal and LPS-induced groups, and those in the downward position correspond to the CN and DXM treatment groups.

The metabolites responsible for the separation between the normal and LPS-induced rats by PC1 (Fig 3B) were identified as lactate, *α*-glucose, malate, choline, creatine/phosphocreatine, glutamate, *ß-*glucose, phosphorylcholine, acetate, taurine, gamma-aminobutyric acid (GABA), pyruvate, glutamine, aspartate, myo-inositol, citrate, choline, glycine, α-ketoglutarate, glycerol, hypoxanthine, and formate. As in S2 Fig B in S1 File, lactate, choline, and acetate were observed to be scattered from other metabolites of the LPS-treated group. These three metabolites were reported previously as potent indicators of hypoxia neuropathy [43].

Hypoxia is a condition wherein the tissue is deprived of adequate oxygen supply, possibly leading to cell necrosis. Frede *et al*. [44] have reported the ability of LPS to induce the hypoxia-inducible factor-1 alpha (HIF-1*α*) in human monocytes and macrophages under normoxic conditions. Induction occurred when the demand for energy supply shifted and the delivery or availability of oxygen in the brain tissue was affected leading to inflammation-associated tissue hypoxia and metabolic acidosis [45].

The elevation of lactate is explicable when local inflammatory activity within the neurovascular unit (NVU) in the brain is caused by the accumulation of extracellular lactate and H^+^, which, in turn, stimulates the peripheral tissue response, known as neurogenic inflammation [46]. Choline is a precursor of the neurotransmitter for acetylcholine. It acts in the same manner as the other neurotransmitters, such as ATP, GABA, and glutamate. Activated microglia express receptors for neurotransmitters and cause an increase in choline and GABA as shown in Fig 3B. Interestingly, the level of one of the important neurotransmitters, glutamate, was lower compared to the normal group. This resembles an earlier finding from this laboratory on the serum metabolites from the same experiment when normal rats were compared with the LPS-induced neuroinflammation group [17]. Based on the synthesis and recycling of glutamate in the TCA cycle, glutamate would then be converted into GABA [47]. This also explains the imbalance of increased or decreased levels of glutamatergic/GABAergic signals as a part of the neuroinflammatory response [48].

The vital metabolic energy for all mammalian cells is glucose. For cerebral functions, about 20% of oxygen and 25% of glucose are consumed by the human body [49]. Glucose and the diffusion of other nutrients into the neural tissue are limited by the restrictive properties of the blood-brain barrier (BBB). As an alternative, glucose is transferred across the extracellular space from the blood through a glucose transporter (GLUT) and a sodium-dependent glucose transporter (SGLT) to the brain [50]. Hence, a wide range of metabolic intermediates, including lactate, pyruvate, glutamate, glutamine, or acetate which are formed from glucose in the brain, can subsequently be oxidized for energy production [51]. These metabolites were detected as the markers in the rats of all the neuroinflammed groups. Other markers of neuroinflammation are the carboxylic acids, formate, and acetate. Formate is a byproduct of acetate production and metabolic acidosis [52]. The increase in both formate and acetate levels results in intracellular acidification within the brain tissue [53]. Brain acidification in the hippocampus of mice due to the LPS-induced neuroinflammation was well-documented by Tyrtyshnaia and colleagues [53].

To understand the possible neuroprotective effect of CN in LPS-induced neuroinflammatory rats, OPLS analysis was carried out between the chemometric integrative data of the ^1^H NMR brain tissue and the quantitative cytokine levels between four selected groups of rats. The groups chosen were the normal rats injected with PBS + water as control (N+water), LPS-neuroinflammed rats + water as control (LPS+water), LPS-neuroinflammed rats treated with aqueous CN at 500 mg/kg of BW (LPS+500CN), and neuroinflammed rats + dextromethorphan (LPS+DXM).

The fold change values, based on the relative quantification of the putative biomarkers from the NMR binned data, are tabulated in Table 2. The table also summarizes the trends between the LPS+DXM group and the LPS+CN groups (500 and 1000 mg/kg) in comparison with both the normal and LPS+water groups, respectively. Despite the variations of biomarkers in the brain tissue samples, the current findings concur with the previously reported results on the sera of the rats from the same experiment [17]. Among the twenty-one detected biomarkers in the LPS-induced neuroinflammed brain tissue, ten shared similar pattern changes as the previously reported results on the serum samples [17]. Lactate, choline, acetate, and formate levels were increased, while pyruvate, creatine, *β*- and *α*-glucose, glutamate, and citrate levels were decreased. The other eleven biomarkers were relatively related to these ten metabolites, as they shared similar metabolic pathways based on the KEGG database. Between the eleven biomarkers are malate, aspartate, citrate and α-ketoglutarate which are involved in the TCA cycle, and glutamine engaged with glutamate and creatine in the amino acid metabolism, while hypoxanthine, taurine, and pyruvate are involved in pyruvate metabolism. Phosphorylcholine, myo-inositol, glycine, and glycerol were in the same group with choline involved with lipid metabolism. Although GABA was detected only in the brain tissue, it was in the chain of the metabolic pathway with glutamate and glutamatergic synapse. The summarized fold changes in Table 2 further established that only five of the twenty-one biomarkers were significantly different based on the modulation of LPS-induction by the CNE treatments (LPS+500CN and LPS+1000CN) when compared with the ten biomarkers for LPS-inducted group treated with dextromethorphan or LPS+water. The differences were also visualized in the cluster pattern of the LPS+500CN, LPS+1000CN, and LPS+DXM groups, being close together, and quite separate from the untreated LPS-induced group (Fig 3A).

**Table 2:** Major biomarkers of LPS neuroinflammed rats and CNE (500 and 1000 mg/kg BW) treatments based on binned data, their fold change values on the 14^th^ day.

	Metabolites	ppm	Fold Change						
			LPS+water /Normal	LPS+DXM/Normal	LPS+500CN /Normal	LPS+1000CN/Normal	LPS+DXM/LPS+water	LPS+500CN/ LPS+water	LPS+1000CN/ LPS+water
**1**	**Lactate**	1.33	+1.42**	-0.97	+1.13	+1.09	-0.68^##^	-0.79^#^	-0.70^##^
**2**	**Pyruvate**	2.47	-0.95*	+0.94	-1.05	-1.03	+1.10^#^	+0.99^#^	+1.08^##^
**3**	**Choline**	4.07	+0.99	+1.04	+1.02	+1.04	+1.05	+1.04	+1.01
**4**	**Malate**	2.37	-1.01	+1.12**	+1.04	+1.04	+1.11^#^	+1.03	+0.99
**5**	**Creatine**	3.04	-1.00	+1.06*	-1.00	+1.02	+1.06^#^	-1.00	+1.00
**6**	α**-Glucose**	3.84	-0.91	-0.92	+1.02	-0.95	+1.01	+1.12^#^	+1.10
**7**	***ß-*Glucose**	3.25	-0.98*	+0.91	+0.96*	+1.05	+1.11^#^	+1.06	+1.07^#^
**8**	**Glutamate**	2.13	-0.98**	+1.08	+1.02	+1.04*	+1.10^#^	+1.04	+1.02
**9**	**Phosphorylcholine**	3.20	-0.92	+1.25	+1.21*	+1.09	+1.37^##^	+1.32^#^	+1.09
**10**	**Acetate**	1.92	+1.07*	+1.13	+1.19*	+1.11*	+1.06	+1.10	+0.93
**11**	**GABA**	2.30	+1.06*	+1.09*	+1.08	+1.04	+1.03	+1.02	-0.94
**12**	**Glutamine**	2.40	-0.95*	+1.05*	-0.95*	+1.03	+1.11^##^	-0.99	+1.05^#^
**13**	**Aspartate**	2.68	-0.97	+1.09*	-1.00	+1.02	+1.13^#^	+1.03	+1.04
**14**	**Myo-inositol**	3.63	+1.00	+1.04*	+1.01	+1.02	-1.04	-1.01	-1.00
**15**	**Citrate**	2.55	+1.02	+1.14*	+1.06*	+1.07*	+1.12^#^	+1.04	+0.98
**16**	**Glycine**	3.57	-0.96	+1.03	-0.98	+1.02	+1.07	+1.02	+1.04
**17**	α**-Ketoglutarate**	2.42	-0.96	+1.03	-0.91*	+1.03	+1.08^#^	-0.95	+1.05^#^
**18**	**Taurine**	3.42	+0.98*	+0.91*	+0.93*	+1.04	+1.12	+1.08	+1.06
**19**	**Glycerol**	3.57	-0.93	-0.92	-0.96	-1.00	+0.98	+1.04	+1.07
**20**	**Hypoxanthine**	8.22	+1.22	+1.30*	+1.27*	+1.19	+1.06	+1.04	-0.82
**21**	**Formate**	8.46	+1.13	-0.84	-0.79	-1.00	-0.75	-0.70	-0.89

The NMR binned data for specific metabolites were analyzed by one-way ANOVA and presented in fold change. The positive and negative values denote an increase and decrease, respectively.

**p<0.001

*p<0.05 with normal, and

##p<0.01 and

#p<0.05 with LPS+water treatment.

#### Integrated data of cytokine levels and ^1^H NMR spectral intensities

Supervised multivariate methods were applied to maximize the variance in the NMR data. Projection to latent structure analysis (PLS) was conducted in combination with ^1^H NMR data-derived spectral information (*X*) and matched cytokine levels (*Y*). To determine the correlation between the metabolic and immune metrics of the cytokines, the incorporation of an orthogonal filter was the best method to be used [54].

The potential biomarkers or the key differential metabolites can be determined by using the OPLS analysis of the ^1^H NMR data of the brain tissue between the normal, LPS+water, LPS+500CN, and LPS+DXM rat groups. Only four out of the initial seven rat groups were chosen to be further examined due to the results of the PCA model showing good clusters for these four treatments. The variable importance in projection (VIP) is the sum over all model dimensions of the contributions with values greater than 1 retained as significant relevant *variables* (S5 Table E in S1 File) [29]. The selected variable (*X*) was then associated with the cytokines expression obtained through microarray quantification data (*Y* variables). Normalization of the work data set for the matrices was ensured through the “transform” page criteria in SIMCA ver.13. The required *X* and *Y* variables were log-transformed as described by Eriksson *et al*. [42], whereby “Min/Max and Skewness” variable values in red were indicators of the required variables to transform. The OPLS model values of R^2^*cum*X = 0.622, R^2^Y = 0.583, Q^2^ = 0.383 showed the goodness of fit and prediction. The permutation tests reconfirmed the validity of the model and fitness of data [29]. In the OPLS permutation plot, the Y axis is the cumulative R2 and Q2 while the X axis is the correlation coefficient between the original *Y* variables and the permuted *Y* variables. The criteria for the validity of the model depend mostly on the R2 and Q2 points (left side) which should be lower than their original points at X = 1 (right side). However, to depend only on observation is unconvincing, thus the regression line is fitted between the two sets of points whereby the Y-intercept of each of the regression lines of R2 and Q2 should not exceed 0.5 and 0.03, respectively [29]. In addition, the R2-line also must be slanting upward, far from becoming a straight horizontal line [42]. All the permutation tests of each Y variable were validated based on the criteria of Y-intercept, wherein all the R2 regression lines were slanted upward (S6 Fig F in S1 File).

The clusters in the OPLS score plot shown in Fig 4A demonstrate the separation between the normal and all of the LPS-induced groups (Water, CNE, and DXM). Covariation between metabolites and cytokines was established; however, the correlations between the relative cytokine levels and metabolites were moderate, as most of the R^2^ values were between 0.6 to 0.313. This might be due to the relatively small group size. The detailed diagrams for the observed versus predicted plots with R^2^ values are provided in S7 Fig G in S1 File.

Fig 4A shows that the LPS+water samples could successfully be distinguished from N+water, LPS+DXM, and most of the LPS+500CN samples by PC1. This suggests that LPS+500CN and LPS+DXM treatments might possess similar effects, as they shared the same metabolite profiles as shown in Fig 4B, 4C and 4E of loading line and scatter plots. The Fig 4B and 4C, respectively provide the metabolite variations of colour-coded according to the value of -1.0 (blue) to 1.0 (red) correlation coefficients of N rows of observation (sample treatment), and K column of variables (metabolites and cytokines expression). The hot coloured signal (red) is an indicator of a more significant contribution to the exhibition of each class separation based on a principal component. Fig 4B derived from PC1, where the upward peaks indicate significant metabolite alterations in the normal rats, and the LPS-induced groups of CN and DXM treatments, while the opposite/downward peaks were noted for the LPS neuroinflammed rats treated with water. Whereas Fig 4C of PC2 revealed the classification between DXM and CN treatment groups. The upward peaks are correlated with the treatment of CN, whereas DXM treatment is in the opposite position. Fig 4B and 4C show similar metabolite distribution with the previous PCA model. As the line plot only contains the metabolite variations, Fig 4D and 4E of the scatter loading plots, combined with both *X*(metabolites) and *Y* (cytokines expression) variables, revealed a better correlation coefficient in the class separation. The plot demonstrates that PC1 positive side, which is related to normal and LPS+CN/DXM treated rats, has better cytokine IL-1*α*, -2, -4, -6, -10, -13, TNF*α* and IFN-*γ* expressions (Fig 4D), with shared metabolites of pyruvate, *α*-glucose, glycerol, *α*-ketoglutarate, taurine, malate, creatine, glutamate, glutamine, phosphorylcholine, aspartate and glycine (Fig 4E). Choline, *β*-glucose, formate, lactate, citrate, GABA, myo-inositol, acetate, and hypoxanthine sharing with the chemokine of MCP-1 and the cytokine, IL-1*β* which belong to the LPS+water treatment (Fig 4E) are located in the negative side of PC1 (Fig 4D).

The similar biomarkers for the treatments of CN500 and DXM could be seen in the biplot of the OPLS model. The overlay of the score and loading plots indicated the distribution of each sample observation cluster pattern with both metabolites and cytokine expression in a single plot (Fig 5). Through PC2, the positive side of LPS+CN500 is revealed to have a higher level of pyruvate, *α*- and *β*-glucose, choline, formate, and glycerol. The cytokines IL-1*α*, -2, -4, -6, -10, -13, TNF*α*, and the chemokine, MCP-1 were also expressed more in the CN treated group. In contrast, lactate, citrate, acetate, hypoxanthine, myo-inositol, GABA, taurine, glutamate, glutamine, *α*-ketoglutarate, glycine, aspartate, malate, phosphorylcholine and creatine, together with the cytokines expression, namely IL-1*β* and IFN*γ*, are higher in the LPS-induced with DXM treatment.

Endotoxin, which is released by the outer membrane of a disrupted Gram-negative bacteria, namely LPS, could trigger diverse mediators of the innate immune system through TLRs, has been well-documented. TLRs can be found in monocytes, macrophages in the brain at the meningeal cells, circumventricular organs, endothelial and perivascular cells, and within the brain parenchyma on microglia and astrocytes [31, 55]. Recent research affirmed the location of TLRs through observation on the circumventricular organs, choroid plexus, meningeal cells, astrocytes, tanycytes, and endothelial cells of the brain by recognition of the LPS binding protein (LBP) and glycoprotein (CD14) mechanism caused by peripheral LPS injection [5]. TLR-4 is one of the LPS native receptors which activates the cascade intracellularly, resulting in translocation of eicosanoids like nuclear factor-*κβ* (NF*κβ*) to the nucleus, whereby the transcription of cytokines occurs. TLR2, with the help of other soluble accessory proteins like myeloid differentiation protein-2 (MD2) and TLR2 incorporated with TLR6, are also able to recognize the bacterial membrane proteins and lipopeptides [6, 7], and act as the LPS receptor.

Various LPS inflammatory cytokines of both pro-inflammatory mediators, such as TNF-α, IL-1*α*, IL-1*β*, and IL-6, and anti-inflammatory mediators, such as IL1r1 and IL-10, have been properly categorized [8, 9, 34, 56]. LPS induction, in combination with IFN*γ* cytokine, is one of the established methods for the *in vitro* induction of inflammation in RAW (macrophage) and BV2 (microglia) cell lines. The MCP-1 chemokine has also been strongly suggested to be involved in the pathogenesis of autoimmune conditions, and is an indicator of gene expression in cancers, asthma, and chronic obstructive pulmonary disease (COPD) [57]. This native inflammatory categorization for the respected cytokines was adopted accordingly in the present study. Other cytokines, such as IL-2, IL-4, and IL-13, which are not generally related to the LPS endotoxin mechanism, were evaluated based on the validated models of OPLS, as depicted in Figs 4 and 5.

Fig 4D shows that the LPS-induced neuroinflammed rat group (LPS+water), with high expressions of IL-1*α*, IL-1*β*, and MCP-1, is separated from the other groups by PC1. The MVDA of the loading scatter plot of this figure exhibits patterns of cytokine regulation which suggest that CN treatment has helped improve the neuroinflammed condition of the rats. The IL-2, -4, -10, and -13 expressions of the anti-inflammatory activities were elevated in the CN-treated groups when compared to the LPS-induced group, as observed by PC1. Even though some pro-inflammatory cytokines, such as TNF-*α*, IFN-*γ* and IL-6 levels in LPS induction were high, the significant reduction of IL-1*β*, IL-1*α*, and MCP-1 suggested a promising ameliorating effect of CN. Pro-inflammatory cytokine IL-6 was found in the normal rats, which might be due to the lesion caused by PBS injection into the rat brains. IL-6 has been deduced as one of the produced mediators in the immediate response to infections and tissue injuries [58].

In brief, the three major types of lymphocytes are B, T, and natural killer (NK) cells. B and T cells are components of the adaptive immune response, while NK cells are for an innate response. The B cells produce antibodies that neutralize the pathogen, while the T cells divide the pathogen into two. The T-helper (Th) cell produces cytokines for immune response, and cytotoxic T cells secrete enzymatic toxic granules for lysis. IL-2 is activated by T cells, whereby it principally helps to maintain lymphoid homeostasis in preventing autoimmune disease [59]. Meanwhile, NK cells activated by IFN cytokine released cytotoxic granules to destroy the infected cells.

IL-2 belongs to the type 1 cytokine receptor family, and IL-4 is a member of type 2 in which they are denoted as T-helper 1(Th1) and 2(Th2), respectively. Th1 produces lymphotoxin-*α* and IFN-*γ*, while Th2 promotes differentiation of naïve CD4 T cells. Generally, IL-4 promotes allergen response to inhibit cell-mediated immune response, which results in macrophage activation and production of pro-inflammatory cytokines [60, 61]. IL-13 shares the same receptor subunits with IL-4, wherein it cooperates in promoting the Th2 response.

Several altered neuroinflammation markers due to CNE treatment could also be observed, as listed in Table 3. The increase of pyruvate in ‘glycolysis and gluconeogenesis metabolism’, and phosphorylcholine in ‘glycerophospholipid and choline’ metabolism was seen in the neuroinflammed brain tissue of the CN-treated rats. Both treatments of CN and DXM lowered the lactate level. The DXM treatment significantly altered several metabolites in the TCA cycle (citrate, malate, aspartate, and α-ketoglutarate), amino acid metabolism (creatine, glutamine, and glutamate), glycolysis and gluconeogenesis (α-glucose and lactate), and the glycerophospholipid and choline metabolism of lipid metabolism (phosphorylcholine). These altered patterns supported the suggestion that CN and DXM shared similar possible ameliorating effects against neuroinflammation in the LPS-induced rat brain. Furthermore, Table 3 summarizes the best four selected signaling molecules among the main altered cytokines, and the significant neuroinflammed biomarkers in brain tissue for normal rats, LPS+500CN, and LPS+DXM. Along with the suggested metabolic pathways involved, LPS-induced rats treated with 5 mg/kg of DXM produced a higher level of Th2, as well as T-helper cells of anti-inflammatory cytokines IL-4 and -10 (Th2), and IL-2 (Th1) when compared with 500 mg/kg of CNE, which only expressed higher levels of IL-2 and -4 of Th1 and Th2, respectively. These treatments showed high pro-inflammatory cytokines of TNF-α and IFN-γ. In contrast to LPS+DXM, which expressed a high level of Th2 helper cells, the LPS+500CN more successfully inhibited IL-1*β*. Based on the cytokine expression, DXM and 500CN treatments were perceived as having a potential ameliorating effect, as both successfully expressed a high level of anti-inflammatory cytokines and decreased the pro-inflammatory cytokines.

**Table 3:** List of the main altered cytokines, the metabolites, and the related pathways.

Group	Altered cytokines^a^	Main metabolic changes in the brain tissue by ^1^H NMR^b^	Related pathway
N+water	↑ IL-2 ↑ IL-10 ↓ IL-1*α* ↓ IL-1*β*	↓ Lactate	Glycolysis/gluconeogenesis, pyruvate metabolism, cAMP signaling pathway
		↑ Pyruvate	Pyruvate metabolism
		↑ *α*-Glucose	Glycolysis/gluconeogenesis
		↑ Glutamate	Glutamatergic/GABAergic synapse in amino acid metabolism. Alanine, aspartate and glutamate metabolism
		↓ Acetate	Pyruvate metabolism, cholinergic synapse
		↑ Glutamine	Glutamatergic/GABAergic synapse. Alanine, aspartate and glutamate metabolism
		↓ GABA	GABAergic synapse
		↓ Taurine	Pyruvate metabolism, Taurine metabolism
LPS+500CN	↑ IL-2 ↑ IL-4 ↓ IL-1β ↑ TNFα	↓ Lactate	Glycolysis/gluconeogenesis, pyruvate metabolism, cAMP signaling pathway
		↑ Pyruvate	Pyruvate metabolism
		↑ Phosphorylcholine	Glycerophospholipid metabolism, choline metabolism
		↑ *α*-Glucose	Glycolysis/gluconeogenesis
LPS+DXM	↑ IL-2 ↑ IL-4 ↑ IL-10 ↑ IFNγ	↓ Lactate	Glycolysis/gluconeogenesis, pyruvate metabolism, cAMP signaling pathway
		↑ Pyruvate	Pyruvate metabolism
		↑ Malate	Citrate cycle (TCA cycle)
		↑ Creatine	Arginine and proline metabolism in amino acid metabolism
		↑ ß -Glucose	Glycolysis/gluconeogenesis
		↑ Glutamate	Glutamatergic/GABAergic synapse. Alanine, aspartate and glutamate metabolism
		↑ Phosphorylcholine	Glycerophospholipid metabolism, choline metabolism
		↑ Glutamine	Glutamatergic/GABAergic synapse. Alanine, aspartate and glutamate metabolism
		↑ Aspartate	Histidine metabolism, Alanine, aspartate and glutamate metabolism
		↑ Citrate	Citrate cycle (TCA cycle)
		↑ α-ketoglutarate	Citrate cycle (TCA cycle)

^a^Altered cytokines: Top four cytokines that co-mapped with the neuroinflammation in the OPLS analysis.

^b^Significant metabolite changes with p value< 0.05 of ANOVA one-way test, ↑ increased, ↓ decreased metabolites, or cytokines when compared to the LPS+water group.

### Integrative analysis of underlying biology

To identify the pathways that were jointly perturbed at the levels of metabolite and cytokine expression, a web tool IMPaLA (Integrated Molecular Pathway Level Analysis; http://impala.molgen.mpg.de/) [62] was utilized. IMPaLA can demonstrate the predictive ability by performing the integrative over-representation analysis for both data matrices of metabolites and cytokines. It also provides a combined p- and q-value that accounts for the significant number of genes and metabolites which are involved in the same biological pathways and processes [63]. The pathways were identified as differentially perturbed by the joint q-value <0.05 as shown in Table 4. This study was established to be well related with the best four processes of the immune system (joint q-value = 1.99x10^-5^), NO-cGMP-PKG mediated neuroprotection (joint q-value = 6.14x10^-5^), signal transduction (joint q-value = 7.55x10^-5^) and HIF-1 (cAMP-PKA) signaling pathway (joint q-value = 5.65x10^-3^).

**Table 4:** Pathways differentially enriched at the metabolomic and genomic levels.

	Process	Pathway source	Overlapping Genes	Gene p-value	Genes q-value	Overlapping Metabolites	Metabolomic p-value	Metabolomic q-value	Joint p-value	Joint q-value
1	Immune System	Reactome	IL1*α*;IL1*β*;IL10;IL2; IL13;IL4;IFN*γ*;IL6; TNF*α*	2.01E-7	1.44E-5	Choline	3.67E-1	1.00	1.28E-6	1.99E-5
2	NO-cGMP-PKG mediated Neuroprotection	Wikipathways	IL1 *β*; IFN*γ;* TNF*α*	5.38E-6	2.97E-4	Glutamate	5.19E-2	3.46E-1	4.49E-6	6.14E-5
3	Signal Transduction	Reactome	IL6; TNF*α*; IL2	3.35E-1	1.00	Glycerol; Acetate; Pyruvate; Glycine; Glutamate; Phosphorylcholine; Formate; GABA	1.09E-6	1.17E-5	5.77E-6	7.55E-5
4	HIF-1 signaling pathway—Homo sapiens (human)	KEGG	IL6;IFN*γ*	2.58E-3	7.95E-2	Lactate	4.87E-2	3.31E-1	1.25E-3	5.65E-3

Overlapping = gene/metabolite is found both in the named pathway and among the identified. p values = significant value for the pathway, q values = the false discovery rate adjusted by correcting the p-value using the Benjamini and Hochberg method.

**Fig 6:**
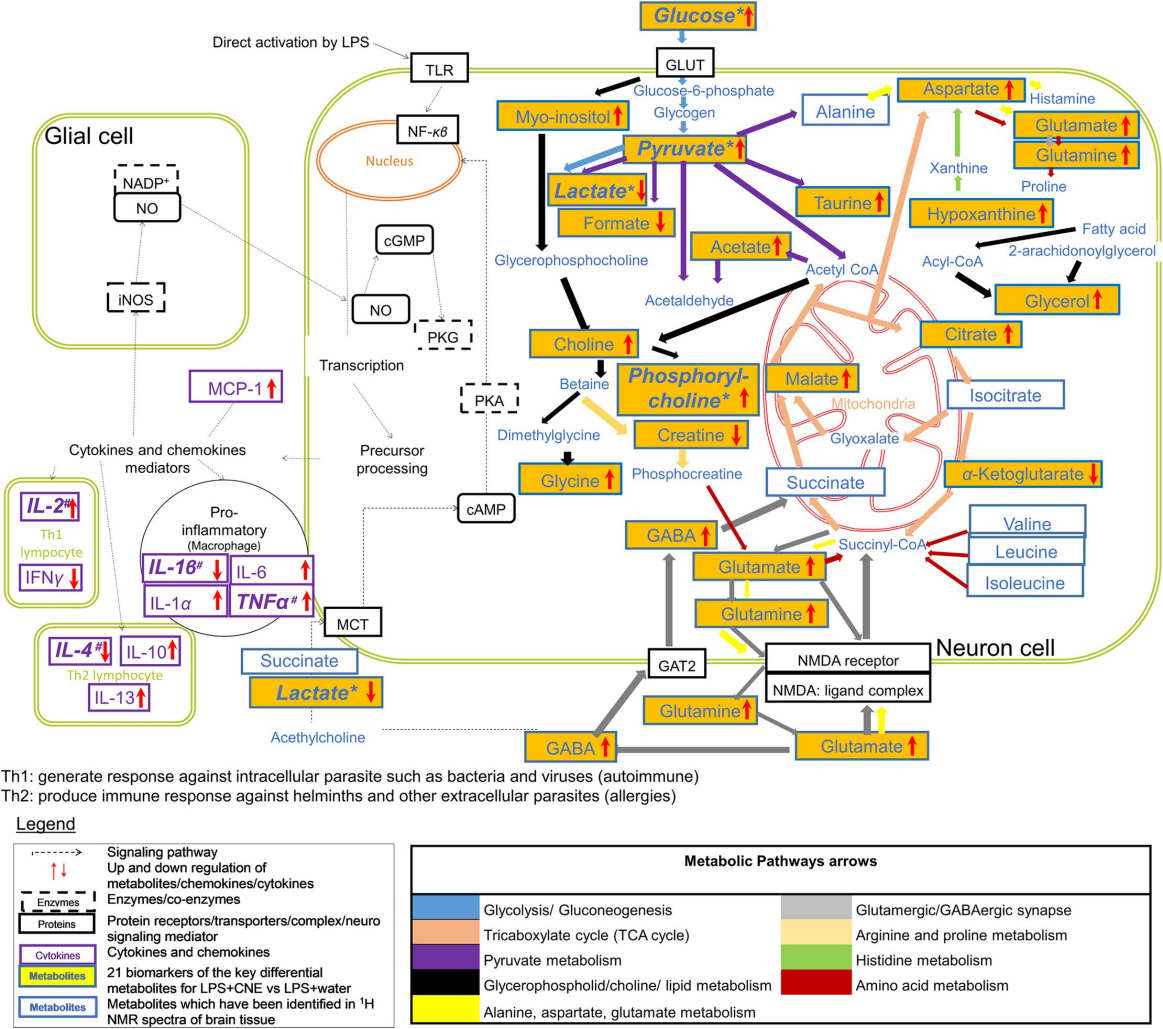
Schematic diagram suggesting the interrelationships of the disturbed metabolic pathways of LPS-induced neuroinflammed rats identified from the ^1^H NMR brain tissue analysis and the cytokine expression analysis. All metabolites are written in blue, whereas purple texts are referred to as cytokines and chemokine. The black texts either represent protein receptor/transporter/complex, enzyme/co-enzyme, or neuro signaling mediator. The dashed arrows represent the signaling pathway and various coloured arrows for metabolic pathways. The metabolites which have been identified in the ^1^H NMR spectra are in blue line boxes, whereas the orange-coloured line boxes are for the 21 suggested biomarkers. The abbreviations for mediators; NO = nitric oxide, IL = interleukin, MCP = monocyte chemoattractant protein, enzymes/coenzymes; iNOs = inducible nitric oxide synthase, NADP^+^ = nicotinamide adenine dinucleotide phosphate, PKG/A = protein kinase G/A, cAMP/cGMP = cyclic nucleotide phosphodiesterases A/G protein kinase, protein receptors/translocators/complex; TLR = toll-like receptor, GLUT = glucose transporter, MCT = monocarboxylate transporter, NF-*κβ* = nuclear factor kappa light chain enhancer activated B cells, NMDA = *N*-methyl-D-aspartate, and GAT = GABA transporter. In the boxes, the up and down red arrows represent the increase and decrease of metabolites/cytokines and chemokine for LPS+CNE treated rats, where * indicates the significant difference of p<0.05 from the one-way ANOVA test, where # indicates the top four in OPLS analysis when compared to LPS+water treatment.

According to the identified pathways involved, Fig 6 displays the suggested schematic diagram depicting the interrelationships of the disturbed metabolic pathways of the LPS-induced neuroinflammed rats with CNE intervention as identified from the ^1^H NMR brain tissue analysis and the cytokine expression.

## Conclusions

The current study revealed the putative anti-neuroinflammatory agents shaftoside, acetate, propionate, alanine, clinacoside C, and 25 additional key chemical markers of CNE against the LPS-induced neuroinflammed rats in reference to the previous *in vitro* LPS-induced microglia study of nitric oxide inhibition [16]. Integrated assessments of the ^1^H NMR metabolites of brain tissue and selected cytokine profiles supported that CNE showed a moderate ameliorating effect against neuroinflammed rats, yet it did not alleviate the level to provide a complete cure. To the best of our knowledge, this is the first comprehensive study of data integration to identify CNE as a potential neuroinflammatory agent targeted on brain tissue metabolic variations. The present data should provide a better platform for future exploration in understanding traditional medicine applications through a comprehensive and holistic approach at the molecular level. However, further investigation with a larger sample size and validation sets need to be developed as they will provide a meaningful biological predictor that can be utilized in further multi-omics research and clinical studies. Such an approach might reveal the true potential of CNE as a natural therapeutic anti-inflammatory agent for nutraceutical and functional food industries.

## Supporting information

S1 File (DOCX)
