# Supplementary material for: The anti-neuroinflammatory effects of Clinacanthus nutans leaf extract on metabolism elucidated through 1H NMR in correlation with cytokines microarray
Source: PLoS One. 2020 Sep 14;15(9):e0238503. doi: 10.1371/journal.pone.0238503 (PMC7489527; doi:10.1371/journal.pone.0238503)
Supplement: S1 File — (DOCX) [file pone.0238503.s001.docx]

**Supporting information**

All of the raw data mentioned in the project can be accessed at:

Private URL: [https://figshare.com/s/7bcb4e38bda161617b7b](https://figshare.com/s/7bcb4e38bda161617b7b),

while the DOI: 10.6084/m9.figshare.12478763 will be public after the article is published.

The Excel workbook, containing 3 sheets of:

-NMR raw data (for PCA and OPLS model)

-Cytokines quantification (for Fig. 1)

-Key metabolites raw data (for fold change in Table 2)

**
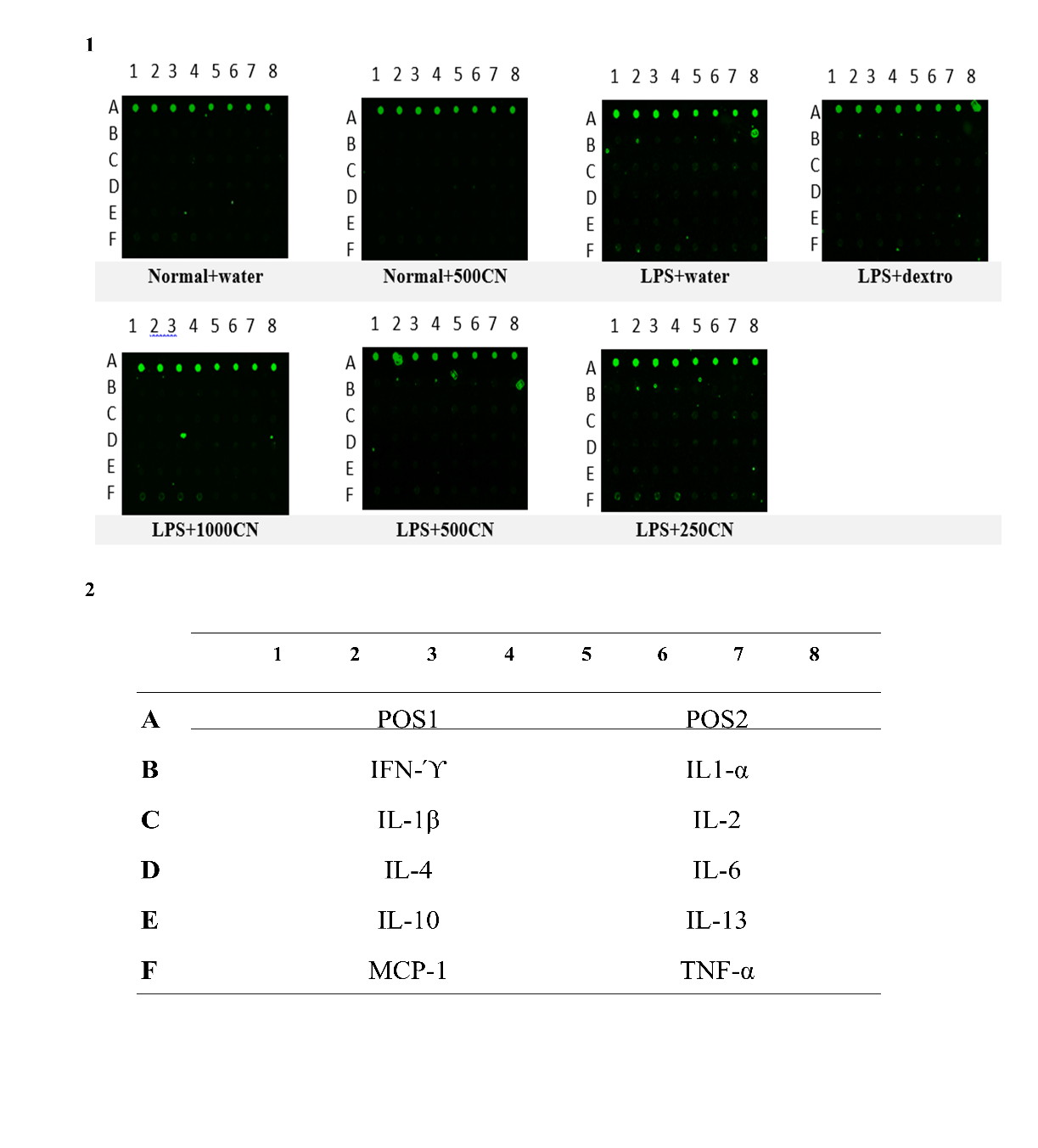
**

**S1 Fig A. Quantitative measurement of ten rat cytokines. (1), Cytokines in the brain cell tissue lysate extracts of the representative samples of Normal+ water, Normal+500CN, LPS+water, LPS+DXM, LPS+1000CN, LPS+500CN, and LPS+250CN rats were detected using cytokine-based arrays. (2), Quantibody array map.** The locations of the cytokine antibodies, positive controls (POS) interferon-gamma (IFN-ϓ), interleukin 1 alpha (IL-1α), interleukin 1 beta (IL-1β), interleukin (IL) -2, -4, -6, 10, -13, tumor necrosis factor-alpha (TNF-α), and monocyte chemoattractant protein-1 (MCP-1) are indicated on the array.

**
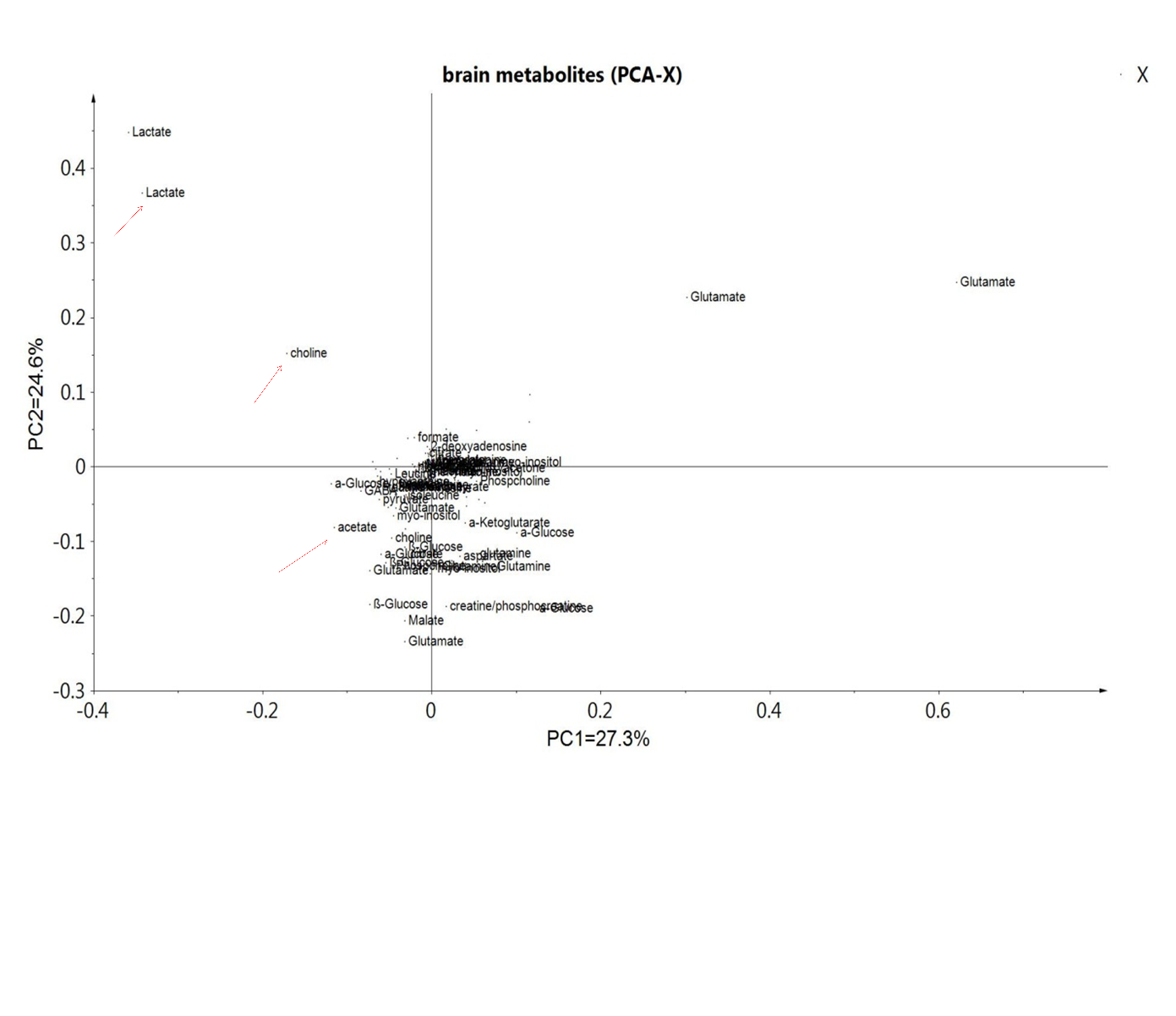
**

**S2 Fig B.** **PCA loading scatter plots of 14^th^ day after CNE treatment based on ^1^H NMR data of rat brain tissue samples.** The red arrow shows the most three discriminative key metabolites which are lactate, citrate, and acetate which possible in inducing hypoxia conditions in LPS treated rats.

**S3 Table C. ^1^H and ^13^C NMR characteristic signals of putative and tentative metabolites in brain tissue extract.**

| Keys | Metabolites | *𝛿* ^1^H (multiplicity)^a^ | *J*-resolved (Hz) | *𝛿* ^13^C, HMBC |
| --- | --- | --- | --- | --- |
| 1 | Isoleucine | 0.94 (t)  1.48 (m)  1.95 (m)  3.67 (d) | 7.5  -  -  4.1 | -  -  -  18.2 |
| 2 | Leucine | 0.96 (t)  1.70 (m),  3.74 (t) | 5.9  -  - | -  -  - |
| 3 | Valine | 0.99 (d)  1.05 (d)  2.28 (m)  3.62 (d) | 7.0  7.0  -  4.3 | 12.2  -  -  - |
| 4 | 3-Hydroxybutyrate | 1.20 (d)  2.34 (m)  4.12 (m) | 6.3  -  - | 24.5  45.1  - |
| 5 | Lactate | 1.33 (d)  4.11 (q) | 7.0  - | 23.0  71.5 |
| 6 | Alanine | 1.48 (d)  3.79 (q) | 7.1  7.2 | 19.2  53.6 |
| 7 | Lysine | 1.48 (m)  1.73 (m)  3.03 (t)  3.76 (t) | -  -  -  6.1 | -  -  -  63.1 |
| 8 | GABA | 1.91 (m)  2.30 (t)  3.02 (t) | -  7.4  7.6 | -  181.5  178.2 |
| 9 | Acetate | 1.92 (s), | - | 26.3 |
| 10 | Glutamate | 2.13 (m)  2.35 (m)  3.76 (dd) | -  -  7.1 | -  -  - |
| 11 | Glutamine | 2.14 (m)  2.45 (m)  3.78 (t) | -  -  6.2 | 29.6  -  57.3 |
| 12 | Malate | 2.37 (dd)  2.68 (dd)  4.31 (dd) | 15.4  -  10.1 | 45.8  -  73.4 |
| 13 | *α*-Ketoglutarate | 2.45 (t)  3.01 (t) | 6.9  6.9 | -  - |
| 14 | Citrate | 2.55 (d)  2.67 (d) | 17.0  17.1 | -  - |
| 15 | Aspartate | 2.68 (dd)  2.82 (dd)  3.91 (dd) | 17.5  3.7  - | 39.6  39.6  - |
| 16 | Creatine/phosphocreatine^b^ | 3.04 (s)  3.94 (s) | -  - | 39.8  56.7 |
| 17 | Choline | 3.21(s)  3.52 (t)  4.07 (m) | -  5.2  - | -  67.8  - |
| 18 | Myo-inositol | 3.29 (t)  3.55 (dd)  3.63 (dd)  4.07 (t) | 9.3  -  9.7  2.8 | -  -  -  - |
| 19 | Taurine | 3.27 (t)  3.42 (t) | 6.5  6.6 | -  - |
| 20 | *β*-Glucose | 3.25 (t),  3.41 (dd)  3.47 (m)  4.65 (d) | 9.8  -  -  8.0 | 96.2  -  -  103.3 |
| 21 | *α*-Glucose | 3.54 (dd)  3.42 (t)  3.84 (m)  5.24 (d) | 9.8  -  -  3.5 | 103.1  -  -  92.2 |
| 22 | Glycerol | 3.57 (m)  3.79 (m) | -  6.5 | -  - |
| 23 | Phosphorylcholine | 3.23 (s)  3.60 (t) | -  5.5 | -  - |
| 24 | Dihydroxyacetone | 4.43 (s), 3.57 (s) | - | - |
| 25 | Glycine | 3.57 (s) | - | 44.5 |
| 26 | Inosine | 6.10 (d)  8.24 (s)  8.35 (s)  4.4 (t) | 5.7  -  -  5.0 | -  -  -  - |
| 27 | Fumarate | 6.53 (s) | - | - |
| 28 | Tyrosine | 6.91 (m), 7.20 (m) | - | - |
| 29 | Phenylalanine | 7.33 (dd)  7.38 (dd)  7.43 (m) | 8.0  7.1  - | -  -  - |
| 30 | Nicotinurate | 8.25 (t), 8.95 (m) | - | - |
| 31 | Hypoxanthine | 8.20 (s), 8.22 (s) | - | - |
| 32 | Formate | 8.46 (s) | - | - |
| 33 | Threonine | 1.33 (d)  3.59 (d)  4.26 (m) | 6.6  5.0  - | 23.0  63.6  - |
| 34 | UDP/UTP^b, c^ | 4.38 (t)  5.99 (d)  7.96 (d) | 5.1  3.0  8.1 | -  -  - |
| 35 | Pyruvate | 2.47 (s) | - | - |
| 36 | Histidine | 7.09 (d)  7.86 (d) | 0.6  1.2 | -  - |
| 37 | Succinate | 2.41 (s) | - | - |
| 38 | Serine | 3.85 (dd)  4.0 (m) | 5.7  - | 59.3  - |
| 39 | 2-Deoxyadenosine | 8.25 (s), 8.34 (s) | - | - |
| 40 | Nicotinate | 8.25 (m)  8.6 (d)  8.9 (d) | -  5.0  2.0 | -  -  - |
| 41 | Trigonelline | 8.82 (m), 9.12(s) | - | - |
| 42 | NADP+ | 9.3 (s)  9.1 (d)  8.82 (d) | -  6.3  8.2 | -  -  - |
| 43 | Anserine | 4.5 (d), 7.1 (d) | - | - |

Multiplicity: s, singlet; d, doublet; dd, double of doublets; t, triplet; q, quartet; m, multiplet; U, unidentified signals; –, signals or multiplicities were not detected. a: Reference to hmdb.ca which accurate to ±0.02 ppm for ^1^H, *J*-resolved ±10 Hz and ±0.5 ppm for ^13^C, b: Tentatively assigned, c: UDP/UTP, uridine diphosphate/uridine triphosphate.

**
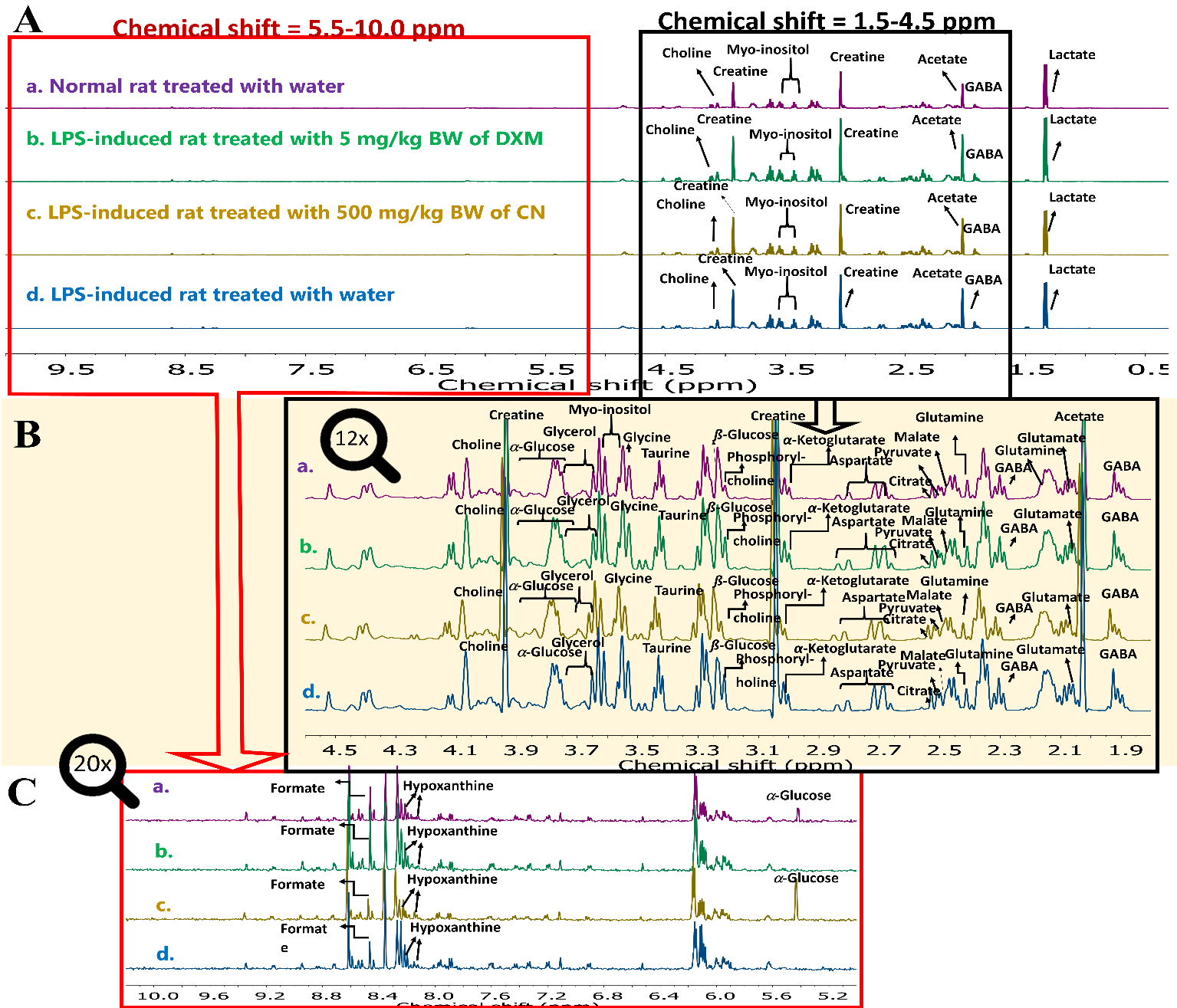
**

**S4 Fig. D. ^1^H NMR representative spectra of the brain tissue: (A) the overall spectra view from 0.5-10 ppm, (B) the 12-fold intensity enlargement on selected spectral within 1.5-4.5 ppm and (C) the 20-fold intensity enlargement in the region of 5.5-10.0 ppm. Each spectrum is a representative of (a)** **normal rats treated with water, (b) LPS-induced rats with 5 mg/kg BW of DXM, (c) LPS-induced rats with 500 mg/kg BW of CN, and (d) LPS-induced rats treated with water after 14 days of treatment.**  Only selected peaks with comparable metabolite intensity are labeled.

**S5 Table E. VIP scores of OPLS model**

| **Chemical shift (ppm)** | **Metabolites** | **VIP scores** | **1.89456 * OPLS cv SE** |
| --- | --- | --- | --- |
| **1.33** | Lactate | 5.073 | 1.255 |
| **3.84** | *α*-Glucose | 4.094 | 2.494 |
| **4.07** | Choline | 3.562 | 2.003 |
| **2.37** | Malate | 2.934 | 1.181 |
| **3.04** | Creatine/phosphocreatine | 2.895 | 0.691 |
| **3.9** | *α*-Glucose | 2.775 | 1.229 |
| **2.13** | Glutamate | 2.645 | 1.075 |
| **3.25** | *β*-Glucose | 2.585 | 1.151 |
| **3.23** | Phosphorylcholine | 2.440 | 0.893 |
| **3.22** | *β*-Glucose | 2.430 | 1.379 |
| **1.92** | Acetate | 2.347 | 0.874 |
| **3.78** | *α*-Glucose | 2.328 | 1.721 |
| **3.42** | Taurine | 1.880 | 0.952 |
| **2.02** | Glutamate | 1.817 | 0.866 |
| **3.5** | *α*-Glucose | 1.675 | 0.702 |
| **2.30** | GABA | 1.626 | 1.249 |
| **2.47** | Pyruvate | 1.596 | 0.691 |
| **3.82** | *α*-Glucose | 1.588 | 0.563 |
| **2.45** | Glutamine | 1.555 | 0.785 |
| **2.68** | Aspartate | 1.454 | 0.473 |
| **3.63** | Myo-inositol | 1.429 | 1.202 |
| **2.55** | Citrate | 1.410 | 0.714 |
| **4.07** | Choline | 1.405 | 0.656 |
| **2.38** | Glutamate | 1.373 | 0.578 |
| **3.57** | Glycine | 1.336 | 0.945 |
| **3.46** | *β*-Glucose | 1.302 | 0.601 |
| **2.45** | *α*-Ketoglutarate | 1.221 | 0.715 |
| **3.57** | Glycerol | 1.106 | 0.427 |
| **8.22** | Hypoxanthine | 1.072 | 0.354 |
| **8.46** | Formate | 1.023 | 1.580 |

VIP: variable importance in projection; OPLS cv: orthogonal partial least squares coefficient of variance; SE: standard error

**
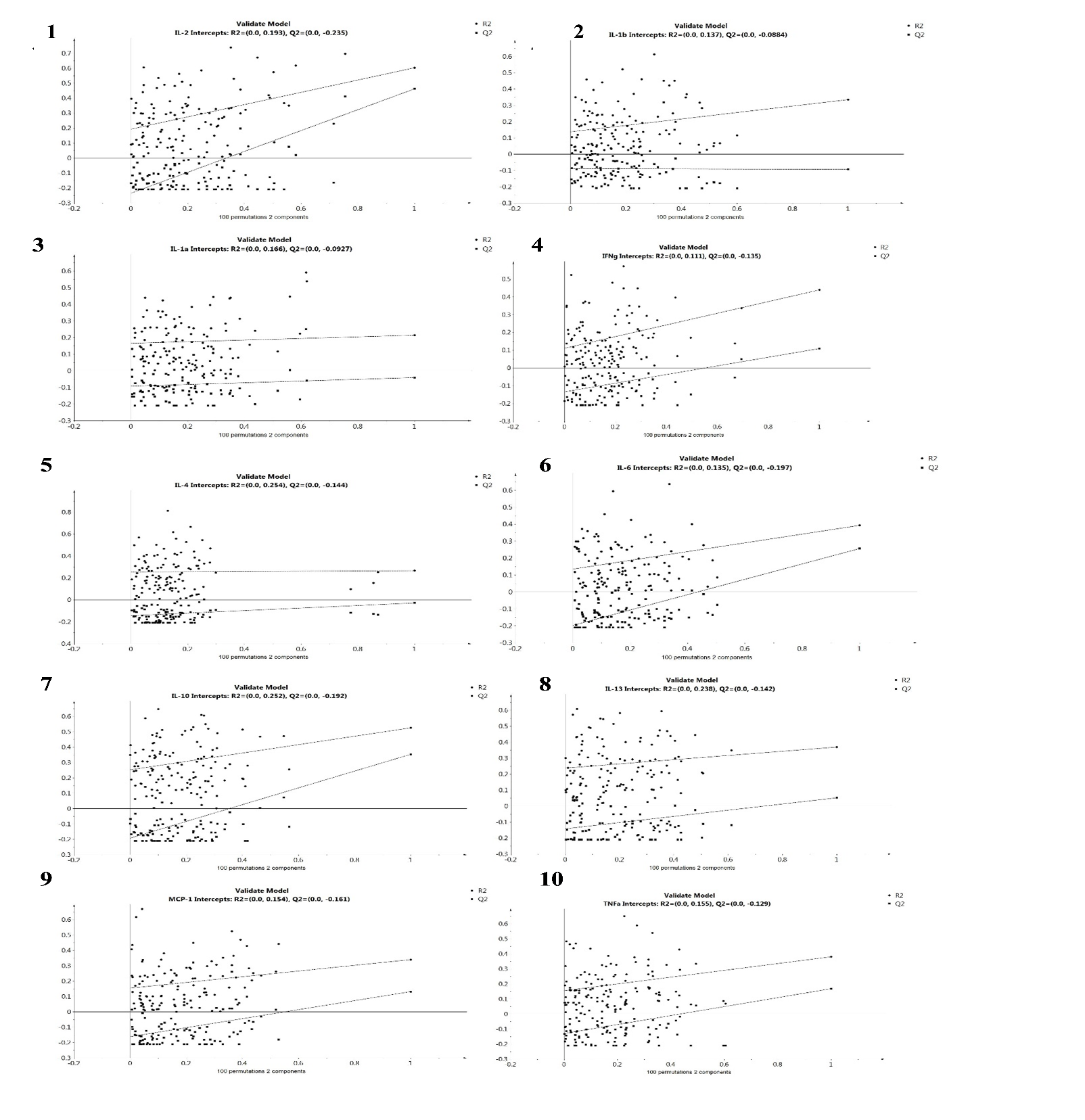
**

**S6 Fig. F. Permutation tests for the OPLS models with Y variables of (1) IL-2, (2) IL-1β, (3) IL-1α, (4) IFN-ϓ, (5) Il-4, (6) Il-6, (7) IL-10, (8) IL-13, (9) MCP-1, and (10) TNF-α.** Each of the variables have R2 and Q2 (R2Y, Q2Y) of Y-intercept at (0.193, -0.235), (0.137, -0.0884), 0.166, -0.0927), (0.111, -0.135), (0.254, -0.1444), (0.135, -0.197), (0.252, -0.192), (0.238, -0142), (0.154, -0.161), and (0.155, -0.129), respectively. All of the models of Y-variables are valid and not overfit.

**
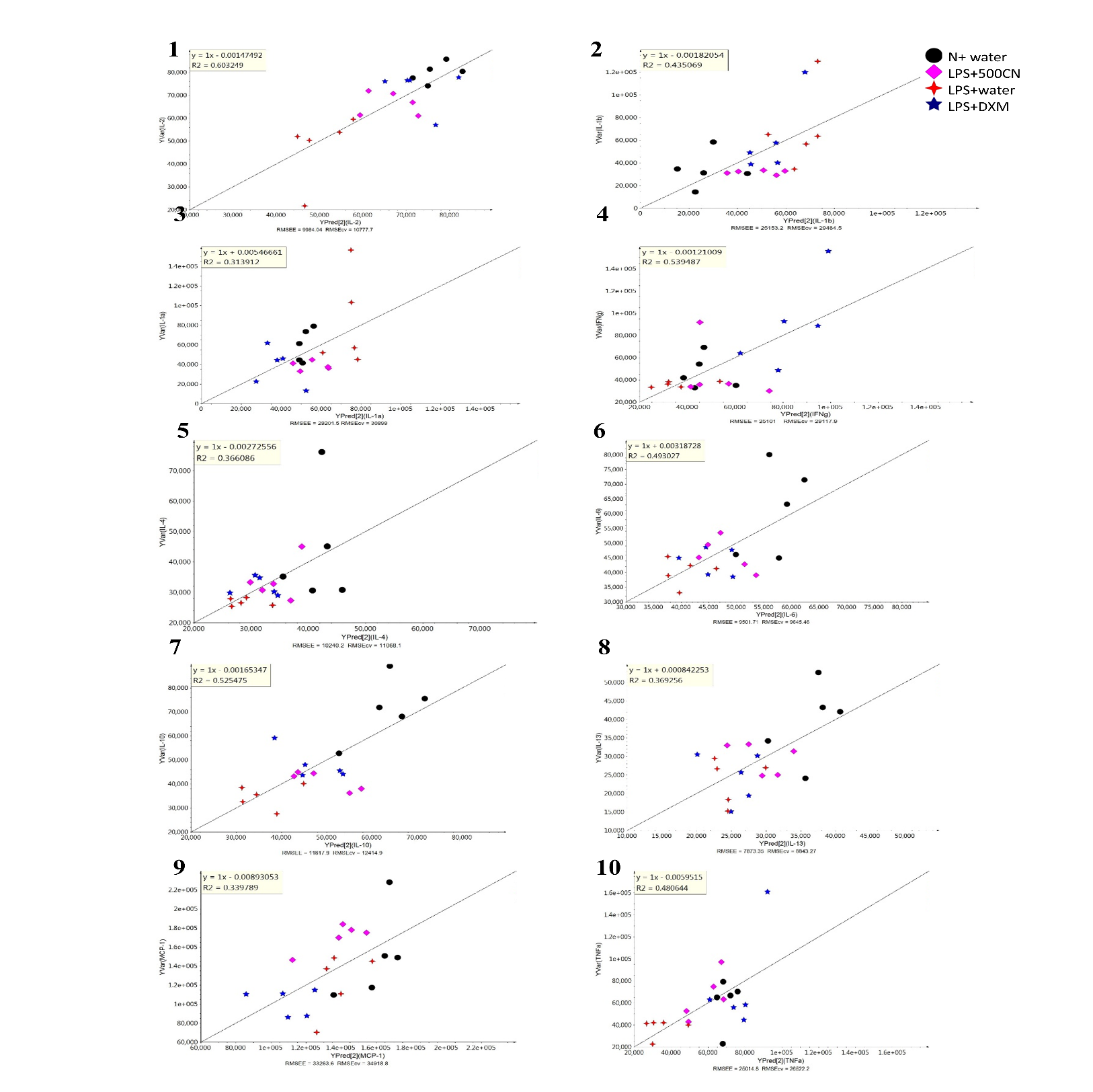
**

**S7 Fig. G**. **Observed vs. predicted plots of (1) IL-2, (2) IL-1β, (3) IL-1α, (4) IFN-ϓ, (5) Il-4, (6) Il-6, (7) IL-10, (8) IL-13, (9) MCP-1, and (10) TNF-α with R^2^ values.** The R^2^ values of the mentioned plots are 0.603, 0.435, 0.313, 0.539, 0.366, 0.493, 0.525, 0.369, 0.339, 0.480, respectively.
